# Isorhamnetin Alleviates Airway Inflammation by Regulating the Nrf2/Keap1 Pathway in a Mouse Model of COPD

**DOI:** 10.3389/fphar.2022.860362

**Published:** 2022-03-24

**Authors:** Yifan Xu, Jing Li, Zhiwei Lin, Weiquan Liang, Lijie Qin, Jiabin Ding, Shuqi Chen, Luqian Zhou

**Affiliations:** ^1^ State Key Laboratory of Respiratory Disease, National Clinical Research Center for Respiratory Disease, Guangzhou Institute of Respiratory Health, First Affiliated Hospital of Guangzhou Medical University, Guangzhou, China; ^2^ Institute of Combination Chinese and Western Medicine, Guangzhou Medical University, Guangzhou, China; ^3^ Department of Respiratory Medicine, The Second People’s Hospital of Foshan, Foshan, China; ^4^ Artemisinin Research Center, Guangzhou University of Chinese Medicine, Guangzhou, China

**Keywords:** isorhamnetin, COPD, inflammation, Nrf2, keap1

## Abstract

Chronic obstructive pulmonary disease (COPD) is a severely disabling chronic lung disease characterized by persistent airway inflammation, which leads to limited expiratory airflow that deteriorates over time. Isorhamnetin (Iso) is one of the most important active components in the fruit of *Hippophae rhamnoides L.* and leaves of *Ginkgo biloba L*, which is widely used in many pulmonary disease studies because of its anti-inflammatory effects. Here, we investigated the pharmacological action of Iso in CS-induced airway inflammation and dissected the anti-inflammation mechanisms of Iso in COPD mice. A mouse model of COPD was established by exposure to cigarette smoke (CS) and intratracheal inhalation of lipopolysaccharide (LPS). Our results illustrated that Iso treatment significantly reduced leukocyte recruitment and excessive secretion of interleukin-6 (IL-6), monocyte chemoattractant protein-1 (MCP-1), and regulated upon activation, normal T-cell expressed and secreted (RANTES) in BALF of CS-induced COPD mice in a dose-dependent manner. This improved airway collagen deposition and emphysema, and further alleviated the decline in lung functions and systemic symptoms of hypoxia and weight loss. Additionally, Iso treatment obviously improves the T lymphocyte dysregualtion in peripheral blood of COPD mice. Mechanistically, Iso may degrade Keap1 through ubiquitination of p62, thereby activating the nuclear factor erythroid 2-related factor (Nrf2) pathway to increase the expression of protective factors, such as heme oxygenase-1 (HO-1), superoxide dismutase (SOD) 1, and SOD2, in lungs of CS-exposed mice, which plays an anti-inflammatory role in COPD. In conclusion, our study indicates that Iso significantly alleviates the inflammatory response in CS-induced COPD mice mainly by affecting the Nrf2/Keap1 pathway. More importantly, Iso exhibited anti-inflammatory effects comparable with Dex in COPD and we did not observe discernible side effects of Iso. The high safety profile of Iso may make it a potential drug candidate for COPD.

## Introduction

Chronic obstructive pulmonary disease (COPD) is a severely disabling chronic lung disease characterized by limited expiratory airflow that deteriorates over time, which eventually leads to typical pathologies of chronic bronchitis and/or emphysema (Brandsma et al., 2020). Patients often present with impaired lung functions, such as a decline in forced expiratory volume (FEV) in 1 s and increases in functional residual and total lung capacities (Tantucci and Modina, 2012). There is ample evidence to suggest that inflammation is a core hallmark of COPD, which plays a major role in the pathological changes in all lung compartments (Brusselle et al., 2011; Decramer et al., 2012; Wang et al., 2018). Cigarette smoke (CS) is the main factor in driving the pathogenesis and progression of COPD (Park and Christman, 2011). CS inhaled into the respiratory tract activates airway epithelial cells and surface macrophages to release multiple inflammatory mediators, particularly chemokines, such as monocyte chemoattractant protein-1 (MCP-1)/CCL2, and regulated upon activation, normal T-cell expressed and secreted (RANTES)/CCL5, which attract circulating neutrophils, monocytes, and lymphocytes into the lungs. Neutrophil and CD8^+^ cell-derived toxic molecules, such as elastase and perforins, contribute to emphysema development and macrophage-derived inflammatory mediators lead to further recruitment of inflammatory cells. These events promote the inflammatory response, thereby forming a feedback loop that promotes persistent chronic inflammation (Larsson, 2008; Barnes, 2016; Jones et al., 2017). Once triggered, the inflammatory reaction of COPD is self-sustaining and continues to progress. Therefore, improving airway inflammation is critical to delay the progression of COPD.

**FIGURE 1:**
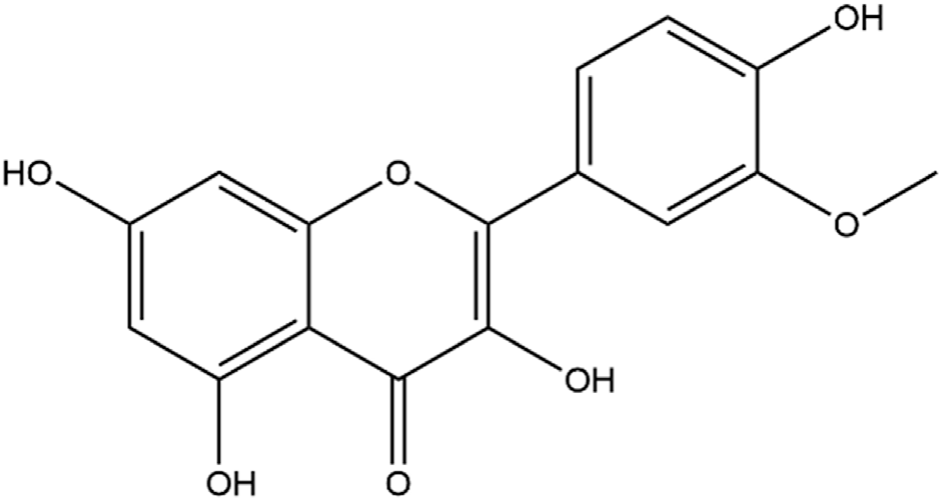
Chemical structure of isorhamnetin.

Current treatments for COPD include bronchodilators, such as long-acting β2-adrenergic receptor agonists and long-acting muscarinic receptor antagonists. However, neither suppress the inflammatory state of the disease without inducing severe adverse effects (Hizawa, 2015). It is recommended that corticosteroid inhalers should be administered only if bronchodilator therapy fails to control symptoms (Wedzicha et al., 2016). Although inhaled and systemic corticosteroids assist in the prevention and treatment of COPD exacerbations, they provide little or no benefit to patients with stable COPD and may have long-term detrimental effects (Barnes, 2010; Zervas et al., 2013). Furthermore, there is evidence that most COPD patients are insensitive to corticosteroid treatments (Tamimi et al., 2012). All of these factors limit the clinical application of corticosteroids. Searching for better COPD therapies requires exploration of new drugs with bioactivities that regulate inflammation and reduce side effects. Many natural products derived from plants have shown significant anti-inflammatory activities *in vitro* and *in vivo* (Prasher et al., 2020; Santana et al., 2016; Yang et al., 2020). Flavonoids are a large group of polyphenolic compounds that are widely found in plants. An increasing number of studies have indicated that flavonoids from traditional Chinese medicine have protective effects against COPD by relieving symptoms and improving lung functions (Yang et al., 2020). Isorhamnetin (Iso), the chemical structure of which is shown in Figure 1, is a natural flavonoid isolated from the fruit of sea buckthorn and ginkgo leaves. It has various benefits that include anti-inflammation, anti-oxidation, and anti-apoptotic properties (Sun et al., 2012; Wang et al., 2018; Li et al., 2021). Additionally, the activity of Iso plays a role in many pulmonary diseases such as acute lung injury, pulmonary arterial hypertension, and influenza (Abdal Dayem et al., 2015; Jnawali et al., 2016; Chang et al., 2020). The mechanism is related to regulating the production of inflammatory mediators, cytokines, and reactive oxygen species (ROS). However, it is unclear whether Iso effectively reduces airway inflammation in COPD.

Among the various signaling pathways, the Nrf2/Keap1 signaling pathway, which mediates the expression of multiple cell-protective genes, plays a major role in regulating airway inflammation in COPD. The nuclear factor erythroid 2-related factor (Nrf2) is an important regulator in controlling both constitutive and inducible resistance to oxidants and electrophiles (Kensler et al., 2007; Cui et al., 2018). While cells are in steady state, Nrf2 is constitutively degraded through binding to Kelch-like ECH-associated protein 1 (Keap1), an adapter protein of E3 ubiquitin ligase. This results in low levels of free Nrf2 protein and represses the transcription of Nrf2-dependent genes. ROS produced by CS exposure induce conformational changes in Keap1 and block its ubiquitination of Nrf2, which results in reduced degradation and rapid accumulation of Nrf2. Then, Nrf2 translocates into the nucleus and forms a heterodimer with one of the small Maf proteins, which binds to the regulatory regions of target genes to upregulate the transcription of antioxidant genes (Zhang et al., 2004; Zhao et al., 2018). Notably, there is growing evidence of anti-inflammatory efficacy of Nrf2 activation in COPD (Sussan et al., 2009; Kobayashi et al., 2016; Jia et al., 2020). Activated Nrf2 inhibits the inflammatory response of COPD by counteracting the inflammation-amplifying effect of ROS (Tian et al., 2021). Moreover, downstream upregulation of the antioxidant gene heme oxygenase-1 (HO-1) inhibits proinflammatory cytokines and activates anti-inflammatory cytokines, thereby promoting inflammatory homeostasis (Wunder and Potter, 2003). Therefore, drugs that target the Nrf2/Keap1 pathway might treat COPD. Our study aimed to verify the anti-inflammatory effect of Iso in a COPD mouse model and to investigate its possible underlying mechanisms.

## Materials and Methods

### Reagents

Iso [≥95% pure (HPLC)] was purchased from Yuanye Bio-Technology Co., Ltd. (B21554, Shanghai, China). Lipopolysaccharide (LPS; L2880) and avertin (T48402) were purchased from Sigma Co. (St Louis, MO, United States). Dex was obtained from Haiwangfuyao Pharmaceutical Co. of Fuzhou (Fuzhou, Fujian province, China). Antibodies are listed in Table 1 **.**

**TABLE 1:** Antibody information.

Antibody	Source/Isotype	Manufacturer	Art. no	Molecular Weight (kDa)
Nrf2	Rabbit	CST	#12721	97/100
Keap1	Rabbit	CST	#8047	60/64
p62	Mouse	Abcam	ab56416	62
HO-1	Rabbit	CST	#43966	28
SOD1	Rabbit	CST	#37385	16/18
SOD2	Rabbit	CST	#13141	22
COX-2	Rabbit	CST	#12282	74

### Animal Model Establishment and Treatments

**FIGURE 2:**
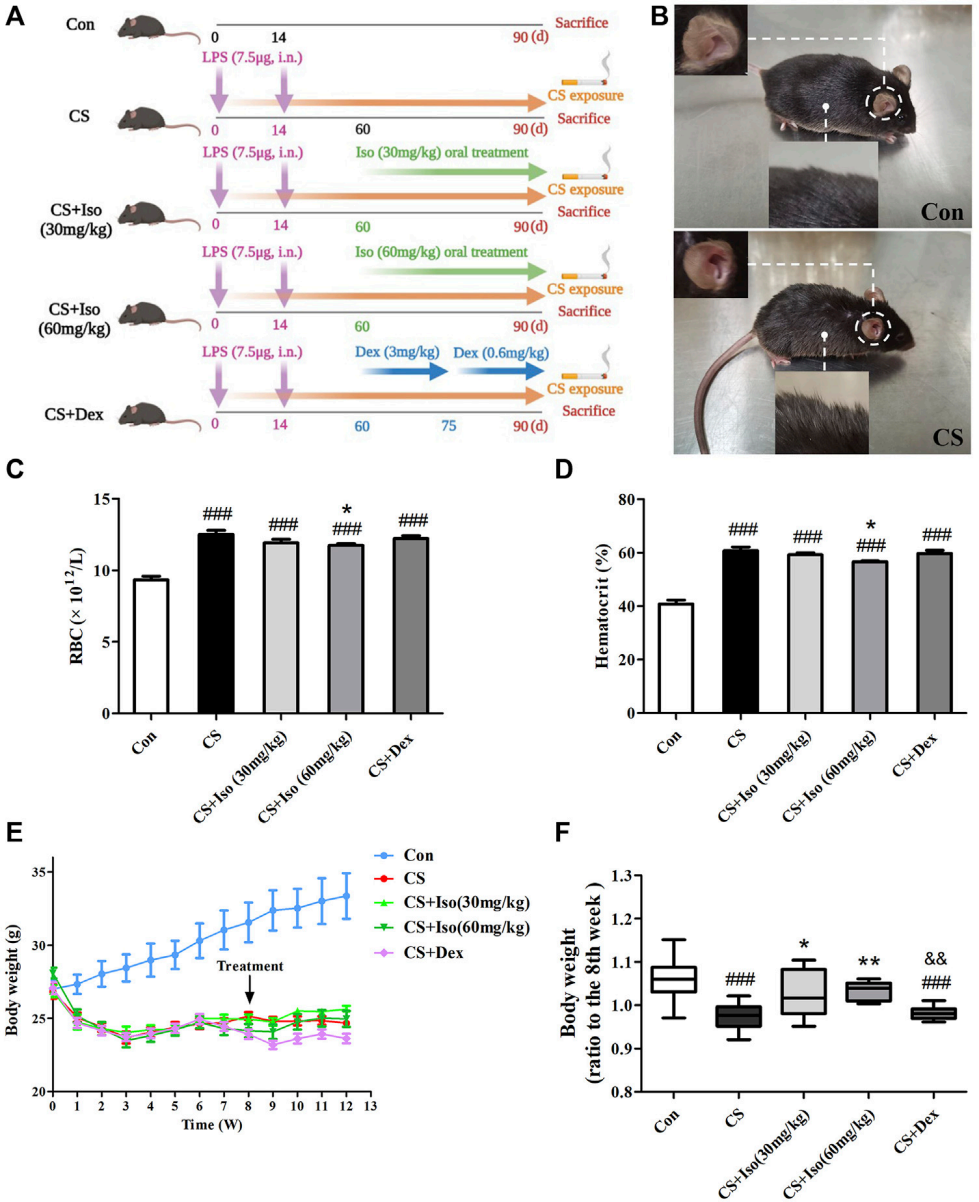
Iso improves CS-induced body weight loss, increases in RBC and HCT in mice. **(A)** The experimental procedure of CS exposure plus LPS instillation as well as Iso or Dex treatment. **(B)** The appearance of COPD mice was significantly different from that of the control group. **(C,D)** The RBC count and hematocrit were measured in whole blood (*n* = 5 mice per group). **(E)** Mouse weight changes during the experiments. **(F)** The improvement of body weight in each group was evaluated (*n* = 15 mice per group). Data was shown as mean (±SD) **(A–E)** and median (±IQR) **(F)**. To test for group differencses one-way ANOVA **(A–E)** and Kurskal-Wallis **(F)** were applied. ^###^ *p* < 0.001 vs. control group, *p < 0.05, **p < 0.01 vs. CS group and ^&&^ *p* < 0.01 vs. CS + Iso 60 mg/kg group.

Accumulating studies have demonstrated that CS exposure and LPS instillation accelerate pulmonary inflammation similar to human COPD (Moazed et al., 2016; Ghorani et al., 2017; Li et al., 2018). Thus, CS exposure and LPS administration were performed in accordance with modified methods described by Li (Li et al., 2018) as shown in Figure 2A. Briefly, healthy wildtype C57BL/6J male mice (6–8 weeks old) were purchased from Liaoning Changsheng Biotechnology. Co., Ltd. (Liaoning, China). Animals were kept in specific pathogen-free facilities with free access to food and water for 1 week before experiments. After the 2 weeks acclimation period, mice were placed in a chamber and exposed to CS (10 cigarettes/1, 2 h/session, twice/day, 6 days/week) from day 0 to 90 except for days with LPS administration. Mice received LPS (7.5 μg/50 μl) by intratracheal inhalation on days 1 and 14. The weight of each mouse was recorded using the same electronic scale every Saturday between 8 and 9 a.m. and weight gain was calculated from the weekly weight data. The cigarettes used in this study were commercial Plum brand cigarettes (Guangdong Tobacco Industry, China). Each cigarette yielded 11 mg tar, 0.9 mg nicotine, and 12 mg carbon monoxide. All experimental protocols were approved by the Committee of Guangzhou Medical University (permit number: 2021044). Seventy-five mice were randomly assigned to the following groups.

Control group: Mice were exposed to room air only and received sterile water by gavage once a day, *n* = 15.

CS group: Mice were exposed to CS (10 cigarettes/1, 2 h/session, twice/day, 6 days/week) from day 0 to 90 and intratracheally administered LPS (7.5 μg/50 μl) on days 1 and 14 without CS exposure, *n* = 15.

CS + Iso (30 mg/kg) group: Mice were exposed to CS combined with intratracheal instillation of LPS and received Iso (30 mg/kg/d) by gavage after day 60, *n* = 15.

CS + Iso (60 mg/kg) group: Mice were exposed to CS combined with intratracheal instillation of LPS and received Iso (60 mg/kg/d) by gavage after day 60, *n* = 15.

CS + Dex group: Mice were exposed to CS combined with intratracheal instillation of LPS and received Dex (3 mg/kg/d for the first 15 days of treatment and 0.6 mg/kg/d for the last 15 days of treatment) by gavage after day 60, *n* = 15.

Finally, all mice were sacrificed on day 91.

### Lung Function Tests

Five mice in each group were anesthetized with 2% avertin (17.5 μl/g i p.), tracheotomized, intubated with a tracheal cannula, and mechanically ventilated using the Forced Pulmonary Maneuver System (Buxco Research Systems, Wilmington, NC, United States) following the manufacturer’s instructions. The average breathing frequency was forcibly set to 150 breathes/min. Lung tissue samples were collected after lung function measurements for further analyses.

### Bronchoalveolar Lavage Fluid Analysis

Five mice in each group were randomly selected for Bronchoalveolar Lavage Fluid (BALF) collection. The left and right lungs were slowly injected with 0.6 mL saline three times with a recovery rate of more than 80%, which was collected in a tube and placed on ice. After centrifugation of the BALF (300 × *g*, 10 min, 4°C), the supernatant was stored at −80°C for subsequent cytokine detection. Then, the cell pellet was resuspended in 1 ml PBS, 10 μl was collected, and total cells were counted using a hemocytometer. The remaining BALF was recentrifuged (300 × *g*, 5 min, 4°C), and the lowest layer of cells was absorbed and evenly smeared on a glass slide. Then, the cells were dried and subjected to Diff-Quik staining (G1542, Solarbio Life Sciences, Beijing, China) for differential counting of neutrophils and macrophages.

### Hematological Analysis

Whole blood samples of five mice in each group were collected by right ventricle puncture at the end of chronic CS exposure. Then, blood samples were processed in whole blood mode using a Sysmex XN-10-B3 hematology analyzer (Sysmex, Japan). The RBC number and hematocrit (HCT) were analyzed.

### Histopathological Examination and Morphological Analysis

The left and right lungs of five mice in each group were slowly perfused with 0.2 ml of 4% paraformaldehyde and then fixed in 4% paraformaldehyde for 24 h. The fixed lung tissues were embedded in paraffin and cut into 4-µm-thick sections for staining with H&E (G1005, Servicebio, Wuhan, China) and Masson trichrome (G1006, Servicebio). The severity of the inflammatory response in lungs was semiquantitatively analyzed by a pathological score defined as: 0 = no detectable inflammation, 1 = mild inflammation with occasional foci of inflammatory cells in bronchial or vascular walls and in the alveolar septa, 2 = moderate inflammation with a one to five cell layer of inflammatory cells in most walls of alveoli, bronchi, or blood vessels, and 3 = severe inflammation with more than five cell layer of inflammatory cells in most walls of bronchi or blood vessels and the alveolar septa (Zhou et al., 2019). Quantification of emphysematous lesions was performed by mean linear intercept (MLI) and mean alveolar area (MAA). Image analysis software Image-Pro Plus 6.0 was used to assess MLI, which is the ratio of the total length (L) of the crossline per field of vision to the number of alveoli (NA) per field of vision intersecting the crossline. The MLI was calculated by the formula:

MLI = L/NA

The airspace surfaces (S) are divided by the number of alveoli (NA) to determine MAA. MAA was calculated by the formula:

MAA = S/NA

Airway thickening was calculated by airway areas divided by their length, which were measured by drawing a line parallel to the airway basement membrane. The Masson-stained sections of lungs were used to assess morphological changes of the amount of collagen deposition. Collagen deposition around the bronchus was assessed by calculating the percentage of the collagen area (blue) in the whole image divided by the total bronchial area using Image-Pro Plus 6.0 software. Five different fields per slide were analyzed, producing one measurement of the degree of airspace enlargement at each of the 5 different distances from the terminal bronchiole. All counts on histology sections were performed by two investigators who were unaware of the treatment protocol of the mouse sections.

### Flow Cytometry

The expression of markers on T cells in blood of five mice in each group was determined by flow cytometry after surface staining with a mouse T lymphocyte subset antibody cocktail, which included anti-CD3, anti-CD4, and anti-CD8 (BD Biosciences, 558431), conjugated with either PE-Cy7, PE, or APC in accordance with standard procedures. Isotype controls were included to ensure correct compensation and confirm antibody specificity. Cells were also stained with FIXABLE VIABILITY DYE EF506 (eBioscience, 65-0866-14) to avoid false positive results. Flow cytometry was performed on a BD FACSVerse flow cytometer and data were analyzed using Flow jo 10.0 software.

### Cytokine and Chemokine Detection

BALF was collected and centrifuged to obtain supernatants from five mice in each group. The concentrations of proinflammatory mediators (IL-6, MCP-1, and RANTES) in BALF were determined using a Bio-Plex Pro Reagent Kit V purchased from Bio-Rad (Cat. #12002798, Minneapolis, MN, United States) following the manufacturer’s instructions.

### Western Blotting

Tissues from three mice in each group were homogenized in ice-cold RIPA lysis buffer (Beyotime Biotechnology, P0013B, China) with phenylmethanesulfonyl fluoride (Beyotime Biotechnology, ST506, China) and then centrifuged to obtain supernatants. The total protein concentration of the supernatants was measured using a bicinchoninic acid protein assay kit (Thermo Fisher Scientific, Inc., Waltham, MA, United States). The same amounts of protein were separated by SDS-polyacrylamide gel electrophoresis and transferred onto a polyvinylidene fluoride membrane. The membranes were blocked with a blocking solution (5% dry skim milk) for 24 h at 4°C. Then, the polyvinylidene fluoride membranes were incubated with specific primary antibodies for 24 h at 4°C, followed by secondary antibodies for 2 h. Finally, the specific proteins were visualized in a luminescent image analyzer and semiquantitative analysis was performed on the basis of the gray value of the band. Protein from one individual animal per group was used for a single WB.

### Statistical Analysis

Experimental data were analyzed using SPSS 25.0 software. The mean ± standard deviation (SD) was adopted for results which passed the normality test and data which did not pass were shown as the median ± interquartile range (IQR). For data not distributed normally, across-group comparison of different groups was shown using the nonparametric Kruskal-Wallis test. For data distributed normally, statistical significance among groups was analyzed by one-way analysis of variance. Tuckey’s post hoc test was performed for multiple comparisons if the population variances were equal and the Games-Howell post hoc test was performed if the population variances were unequal. *p* < 0.05 was considered statistically significant.

## Results

### Iso Improves General Symptoms of CS-Induced Chronic Obstructive Pulmonary Disease Mice

In this model, mice underwent intratracheal inhalation of LPS twice and CS exposure (Figure 2A), which showed typical clinical manifestations of COPD. Compared with the control group, CS group mice presented abnormal symptoms that included shaggy hair, irritability, decreased activity, and even shedding (Figure 2B), which were aggravated as the experiment progressed. Additionally, the mucosal color of mice in the model group was ruddier than that in the normal group, which may be due to compensatory hyperplasia of erythrocytes caused by chronic hypoxia (Haider and Anwer, 2021). Consistent with this, RBCs and HCT were significantly increased in peripheral blood of CS-induced COPD compared with the control group. After administration of Iso, the trends in the above indicators were reversed, especially at a high dose (60 mg/kg). However, Dex administration had little effect on improvement of the RBC number and HCT (Figures 2C,D). In addition to respiratory manifestations, COPD usually presents with systemic manifestations such as weight loss. Similarly, we found that the weight of mice in the CS group was significantly lower than that of mice in the control group, which was apparently ameliorated by Iso at doses of 30 and 60 mg/kg. Interestingly, Dex treatment appeared to have little effect on weight loss associated with CS exposure (Figures 2E,F). We found that treatment with Iso, especially at 60 mg/kg, resulted in a significant difference in the effect on body weight than treatment with Dex.

### Iso Ameliorates Lung Function Decline of CS-Induced Chronic Obstructive Pulmonary Disease Mice

**FIGURE 3:**
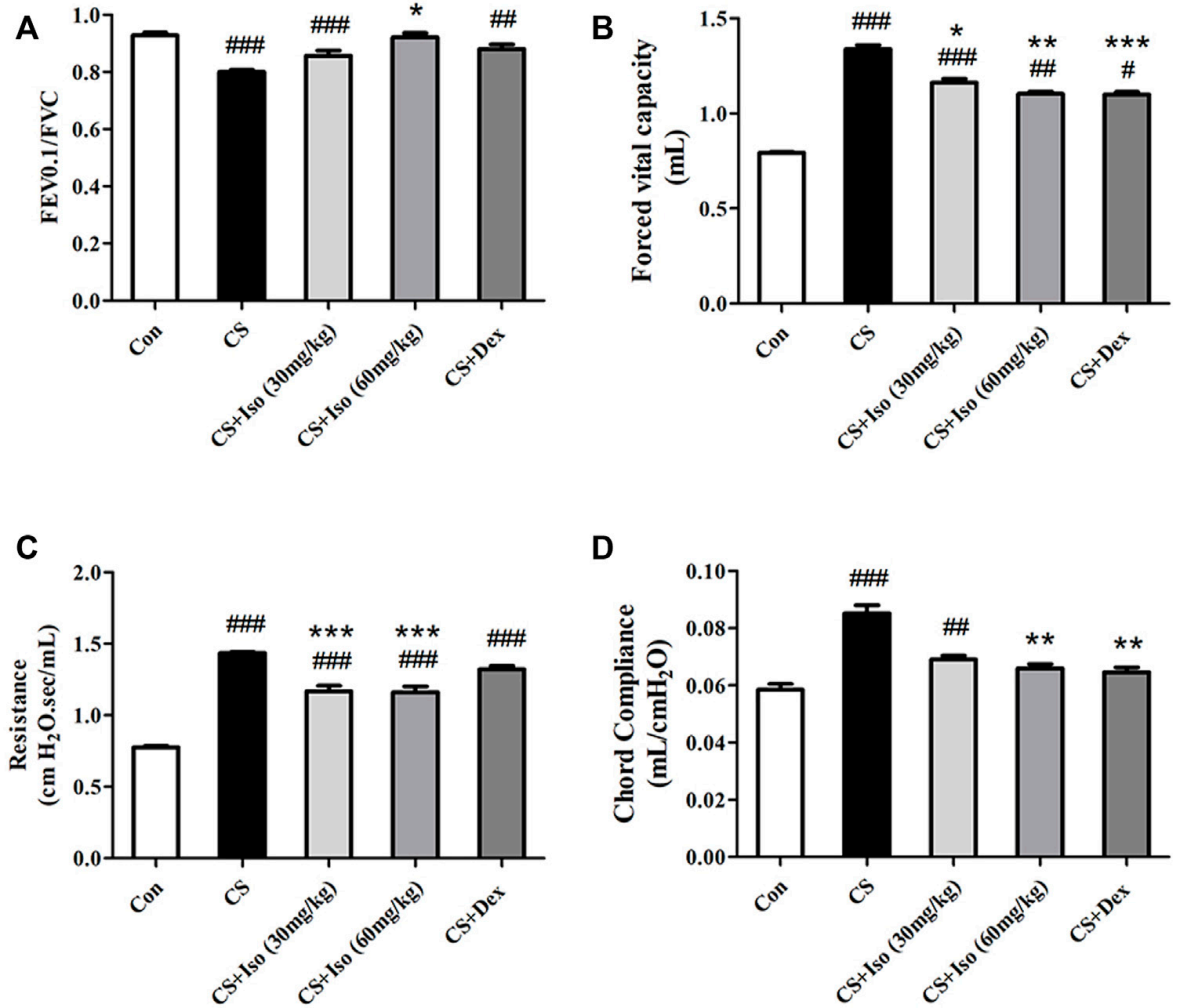
Iso alleviates lung functions decline of CS-induced COPD mice, which was described by increasing **(A)** FEV0.1/FVC and decreasing **(B)** forced vital capacity, **(C)** resistance index and **(D)** chord compliance (*n* = 5 mice per group). Data was shown as mean (±SD) **(A–D)**. To test for group differences one-way ANOVA **(A–D)** was applied. ^#^ *p* < 0.05, ^##^ *p* < 0.01, ^###^ *p* < 0.001 vs. control group and *p < 0.05, **p < 0.01, ***p < 0.001 vs. CS group.

Considering that the small airway dysfunction due to persistent airway inflammation of the lungs and parenchyma decreases lung functions in COPD, we evaluated the effect of Iso on lung functions in CS-exposed mice. Compared with the control group, CS-exposed mice exhibited significant airflow restriction and pulmonary hyperinflation (Figures 3A–C). Additionally, CS increased chord compliance (Cchord) (Figure 3D). Iso at a dose of 30 mg/kg significantly reduced the increase in FVC and RI, and descended the trend of deterioration in FEV0.1/FVC and Cchord caused by CS exposure, while all of the above indicators were significantly improved by Iso at a dose of 60 mg/kg (Figures 3A–D). Additionally, treatment with Dex obviously improved the CS-induced decline in FVC, Cchord, while not significantly improved the FEV0.1/FVC and RI (Figures 3A–D).

### Iso Reduces Emphysema and Infiltration of Inflammatory Cells in the Lungs of CS-Induced Chronic Obstructive Pulmonary Disease Mice

**FIGURE 4:**
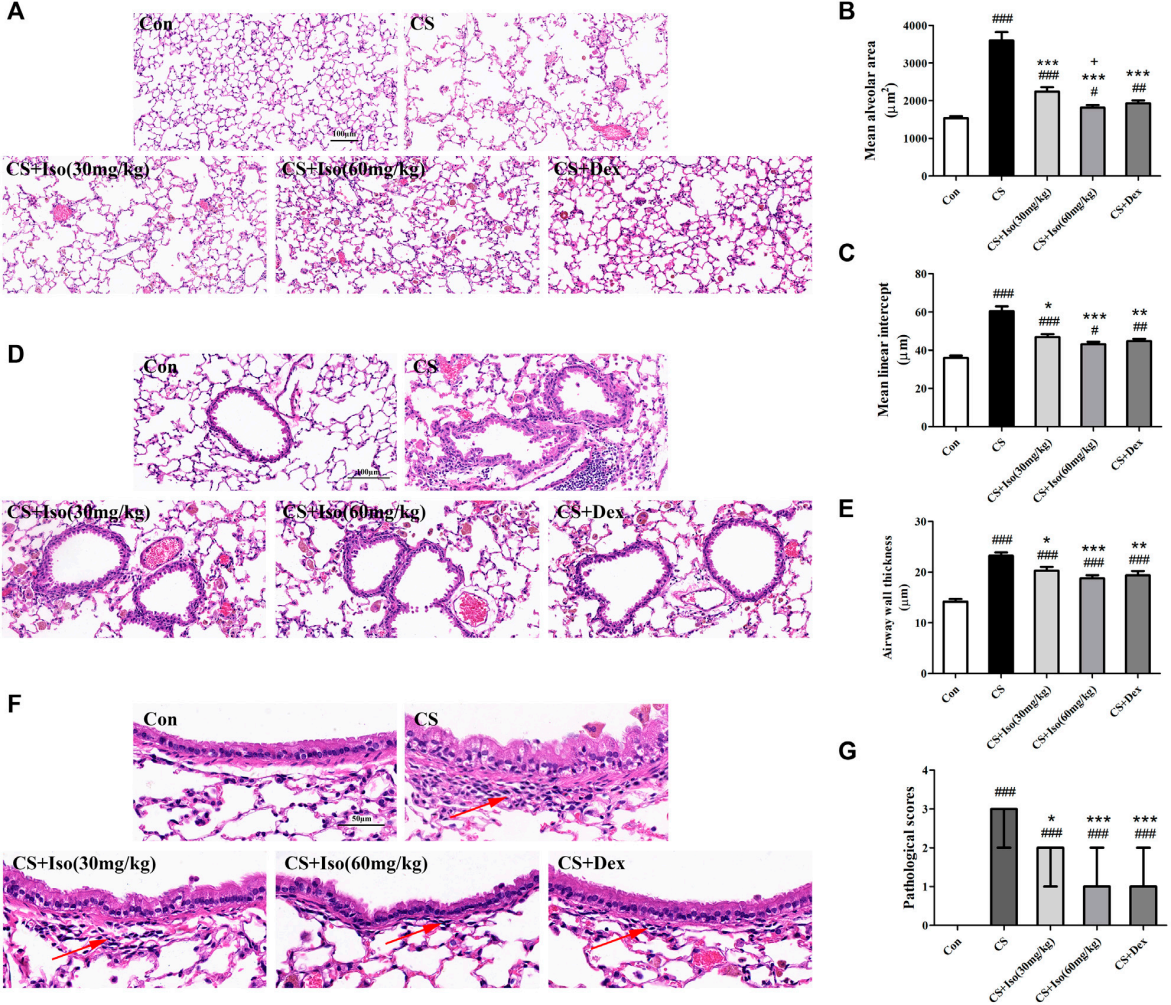
Iso reduces emphysema and infiltration of inflammatory cells in the lungs of CS-induced COPD mice. Lung tissues were subjected to H&E staining. Representative histopathological sections of mouse lungs showed various degrees of **(A)** distensible alveolar spaces (scale bar 100 μm), **(D)** incrassate bronchial walls (scale bar 100 μm) and **(F)** infiltrative inflammatory cells (arrow) (scale bar 50 μm). These lesions were quantified using different indicators for statistical analysis, as seen by the **(B)** mean alveolar area and **(C)** mean linear intercept, which were quantitative analysis of the severity of emphysema (*n* = 5 mice per group). Moreover, quantitative analysis of the degree of inflammation in lungs was determined by **(E)** the airway wall thickness and **(G)** pathological scores (*n* = 5 mice per group). Data was shown as mean (±SD) **(B,C,E)** and median (±IQR) **(G)**. To test for group differencses one-way ANOVA **(B,C,E)** and Kurskal-Wallis **(G)** were applied. ^#^ *p* < 0.05, ^##^ *p* < 0.01, ^###^ *p* < 0.001 vs. control group, *p < 0.05, **p < 0.01, ***p < 0.001 vs. CS group and ^+^ *p* < 0.05 vs. CS + Iso 30 mg/kg group.

To evaluate the regulatory effect of Iso on emphysema and inflammatory cell infiltration into lungs, histopathological examination was performed by H&E staining. Compared with the control group, histopathological analysis of lungs in CS-induced COPD mice revealed dilated alveoli, an impaired alveolar wall, and alveolar wall fusion, a feature in accordance with the pathology of typical emphysema (Figure 4A). As shown in Figures 4B,C, compared with the control group, MLI and MAA were increased in the CS groups. Interestingly, Iso at doses of 30 and 60 mg/kg significantly restrained pulmonary structural destruction caused by CS exposure. Additionally, lung tissues in the CS group showed obvious inflammatory alterations compared with the control group, which manifested as thickening of the bronchial wall, disorder of epithelial cells, and infiltration of a large number of inflammatory cells. Conversely, the above histological damages were improved in mice treated with Iso in a dose-dependent manner (Figures 4D–G). Furthermore, treatment with Dex markedly inhibited the emphysema and infiltration of inflammatory cells of CS-induced COPD, which further substantiated the clinical anti-inflammatory therapeutic effect of this medicine (Figure 4). There was no significant difference between treatment with Iso and Dex inimproving the emphysema and infiltration of inflammatory cells.

### Iso Attenuates Collagen Deposition in the Airways of CS-Induced Chronic Obstructive Pulmonary Disease Mice

**FIGURE 5:**
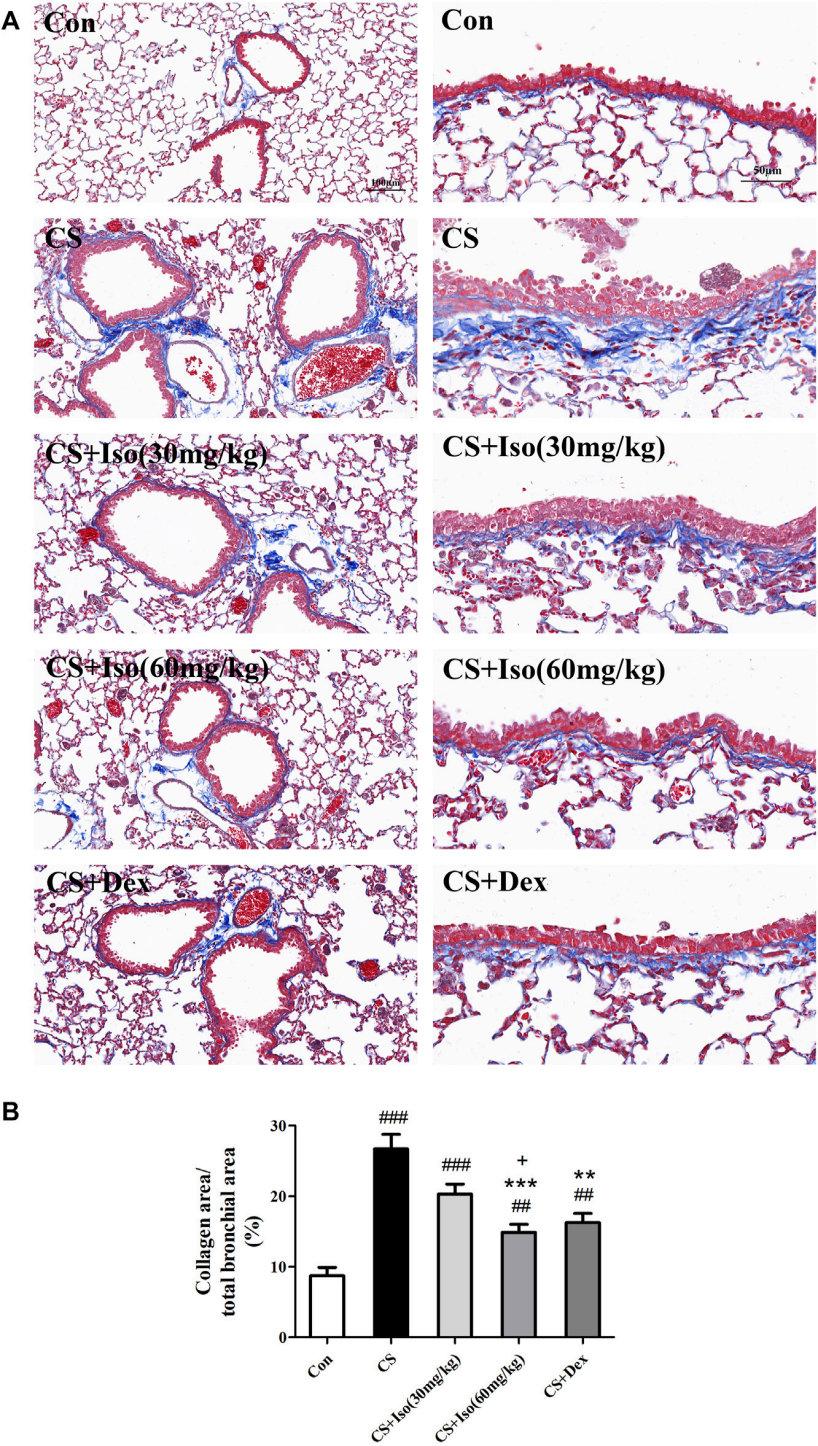
Iso inhibits the collagen deposition induced by CS. Lung tissues were subjected to Masson staining. **(A)** Representative histopathological sections of collagen deposition (blue color) in lungs (scale bar 100 μm/50 μm). **(B)** Quantitative analysis of collagen deposition (*n* = 5 mice per group). Data was shown as mean (±SD) **(B)**. To test for group differencses one-way ANOVA **(B)** was applied. ^##^ *p* < 0.01, ^###^ *p* < 0.001 vs. control group and **p < 0.01, ***p < 0.001 vs. CS group and ^+^ *p* < 0.05 vs. CS + Iso 30 mg/kg group.

Persistent airway inflammation in COPD leads to deposition of collagen fibers around the airway, which reduces lung elasticity and further exacerbates COPD (Han et al., 2021). To determine the effect of Iso ondsla CS-induced fibrosis, lung sections were stained with Masson trichrome. As shown in Figure 5, in lung tissue of control group mice, only little collagen deposition was detected around vessels and bronchioles. However, extensive collagen was readily observed in lung tissue of CS group mice compared with the control group, which confirmed CS-induced fibrosis in lung tissue (Figure 5). After Iso treatment, a significant decrease in collagen deposition was observed around the airway wall compared with the CS group, especially at a high dose (60 mg/kg). Additionally, an obvious reduction in collagen deposition was observed around vessels and bronchioles after Dex treatment. We observed no significant difference between Iso and Dex in the improvement of pulmonary collagen deposition in COPD mice.

### Iso Reduces Recruitment of Leukocytes and Airway Inflammation of CS-Induced Chronic Obstructive Pulmonary Disease Mice

**FIGURE 6:**
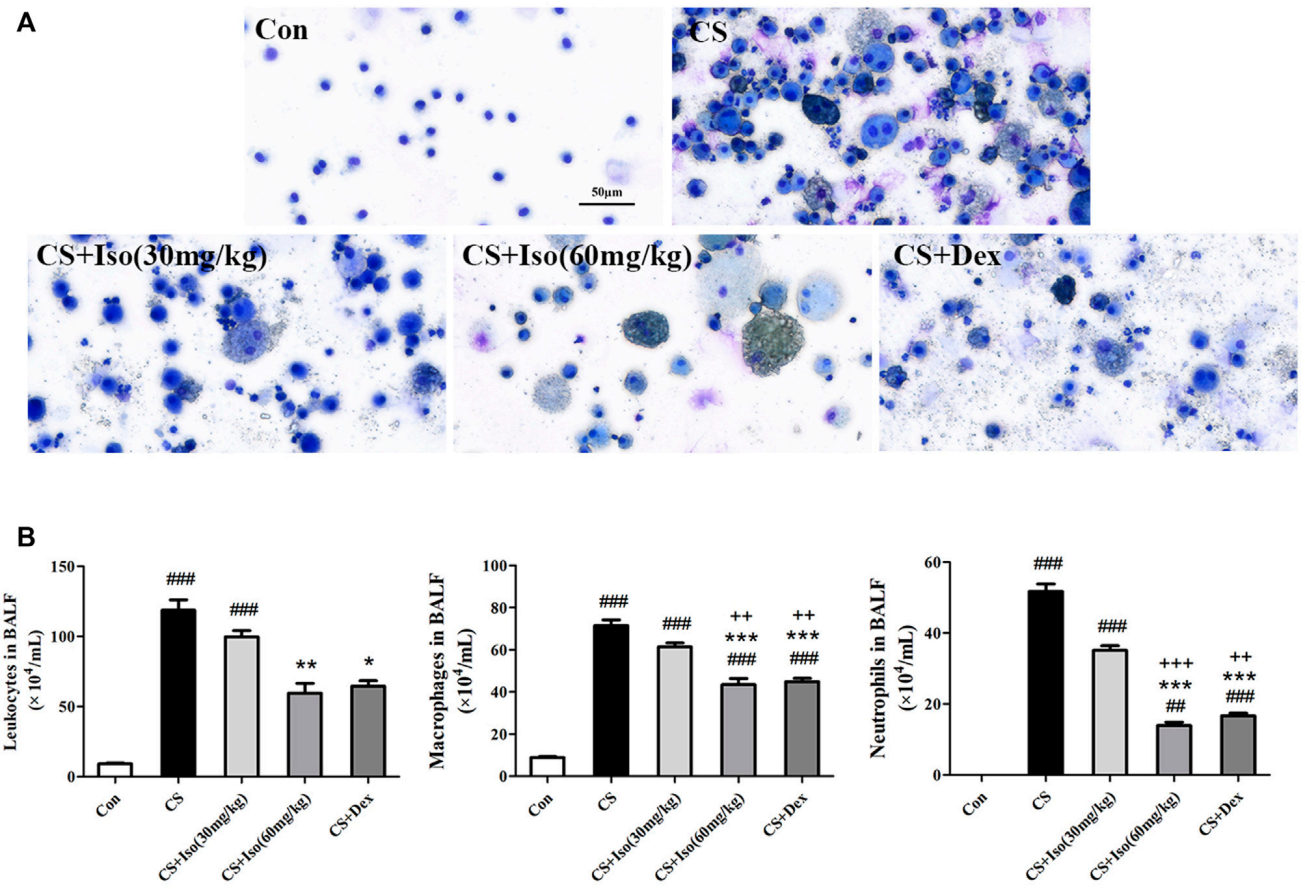
Iso inhibits the recruitment of neutrophils and macrophages in the BALF of CS-induced COPD mice. Leukocytes in BALF were subjected to Diff-Quik staining. **(A)** Representative images of leukocytes in BALF from each group (scale bar 50 μm). **(B)** The numbers of total cells, macrophages and neutrophils in BALF (*n* = 5 mice per group). Data was shown as mean (±SD) **(B)**. To test for group differencses one-way ANOVA **(B)** was applied. ^##^ *p* < 0.01, ^###^ *p* < 0.001 vs. control group, *p < 0.05, **p < 0.01, ***p < 0.001 vs. CS group and ^++^ *p* < 0.01, ^+++^ *p* < 0.001 vs. CS + Iso 30 mg/kg group.

To investigate the regulatory effect of Iso on airway inflammation, BALF was collected to count inflammatory cells and analyze inflammatory factors. First, total cells were analyzed in the BALF of each group. We found that the total number of leukocytes in BALF of the CS group was significantly higher than that in the control group, whereas Iso at a dose of 60 mg/kg apparently reduced the numbers of leukocytes in BALF of the CS group. Next, leukocytes in BALF from each group were classified and counted as shown in Figure 6. It appeared that the cells in BALF of the control group were almost entirely composed of macrophages. However, CS triggered the recruitment of a large number of neutrophils and macrophages with engulfing soot particles. However, the increase in macrophages and neutrophils induced by CS was downregulated by treatment with Iso in a dose-dependent manner, especially at a high dose (60 mg/kg). Moreover, Dex treatment also showed marked efficacy to inhibit leukocyte recruitment. The regulatory effect of Iso at a dose of 30 mg/kg on macrophages and neutrophils in BALF appeared less than that of 60 mg/kg, but there was no significant difference between treatment with Iso at a dose of 60 mg/kg and Dex in improving the accumulation ofleukocytes.

**FIGURE 7:**
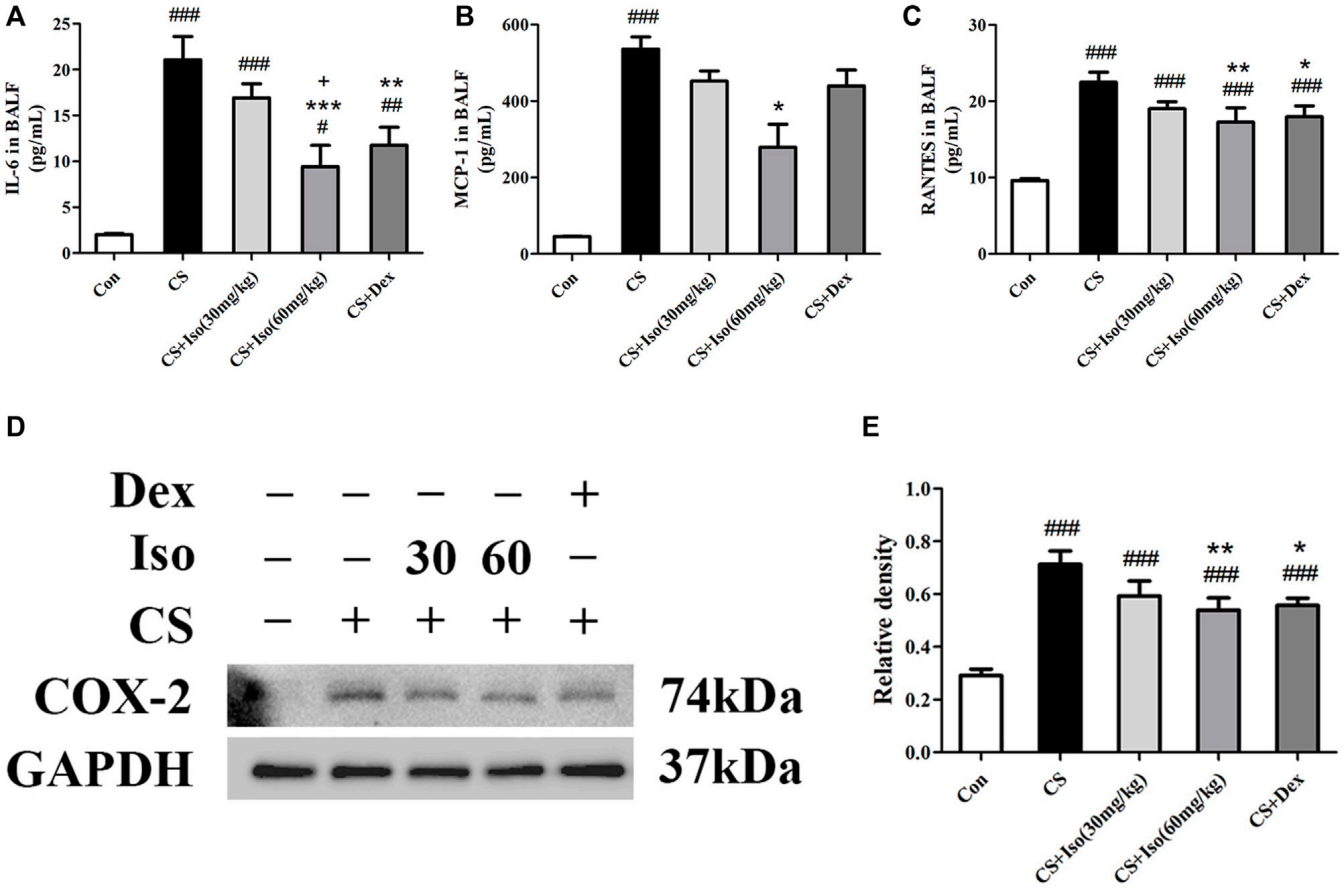
Iso reduces the excessive expression of proinflammatory cytokines induced by CS. **(A–C)** The levels of IL-6, MCP-1 and RANTES in BALF were measured (*n* = 5 mice per group). **(D)** The protein expression level of COX-2 in lung tissues was analyzed using Western blotting (*n* = 3 mice per group). **(E)** The band intensity of COX-2 was semiquantified using ImageJ. GAPDH was used as the internal reference. Data was shown as mean (±SD) **(A–C–E)**. To test for group differencses one-way ANOVA **(A–C–E)** was applied. ^#^ *p* < 0.05, ^##^ *p* < 0.01, ^###^ *p* < 0.001 vs. control group, *p < 0.05, **p < 0.01, ***p < 0.001 vs. CS group and ^+^ *p* < 0.05 vs. CS + Iso 30 mg/kg group.

We then analyzed the effects of Iso on cytokine secretion in CS-exposed mice. Consistently, Iso at a dose of 60 mg/kg markedly reduced CS-induced production of IL-6, MCP-1, and RANTES in BALF (Figures 7A–C). Additionally, we detected the expression of inflammatory mediator Cyclooxygenase-2 (COX-2) in lung tissues, which was significantly upregulated in the CS group compared with the control group and downregulated by Iso in a dose-dependent manner, especially at a high dose (Figures 7D,E). Dex also reduced the excessive production of IL-6 and RANTES in BALF and COX-2 in lung tissues, but had little effect on the expression of MCP-1 (Figure 7).

### Iso Regulates T Lymphocytes in Peripheral Blood of CS-Induced Chronic Obstructive Pulmonary Disease Mice

**FIGURE 8:**
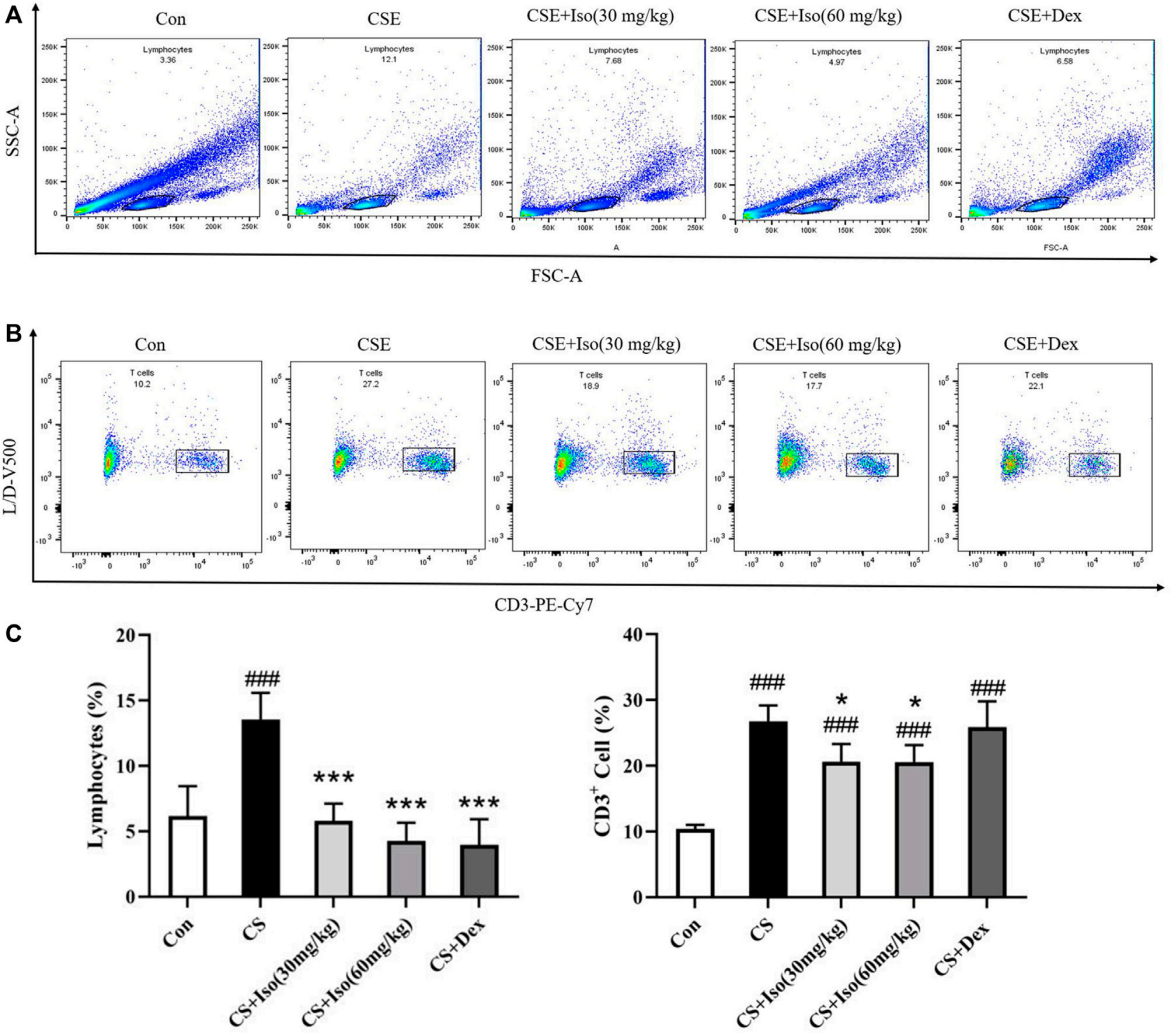
Iso regulates T lymphocytes in peripheral blood. The expression of markers on T cells in blood was determined by flow cytometry after surface staining with the anti-mouse specific Abs conjugated with either PE-Cy7, PE, or APC. **(A)** Lymphocytes were identified based on their characteristic properties shown in by forward scatter area (FSC-A) and side scatter area (SSC-A). **(B)** CD3^+^ lymphocytes were identified based on their characteristic shown in PE-Cv7-A, and **(C)** the proportions of lymphocytes and CD3^+^ lymphocytes in whole blood were calculated separately (*n* = 4 mice per group). Data was shown as mean (±SD) **(C)**. To test for group differencses one-way ANOVA **(C)** was applied. ^###^ *p* < 0.001 vs. control group, *p < 0.05, ***p < 0.001 vs. CS group.

It has been reported that T lymphocytes play major and diverse roles in the establishment of inflammation in COPD (Gupta et al., 2007; Li et al., 2021). To evaluate the regulatory effect of Iso on T lymphocytes, we investigated the proportions of lymphocytes and CD3^+^ lymphocytes in whole blood separately. CS triggered an obvious increase in the lymphocyte ratio and CD3^+^ lymphocyte ratio in blood compared with the control group. Furthermore, the above alterations were markedly ameliorated by Iso at doses of 30 and 60 mg/kg. Dex significantly attenuated the proportion of lymphocytes but had no effect on CD3^+^ lymphocytes (Figure 8).

### Iso Regulates the Nrf2/Keap1 Pathway in CS-Induced Chronic Obstructive Pulmonary Disease Mice

**FIGURE 9:**
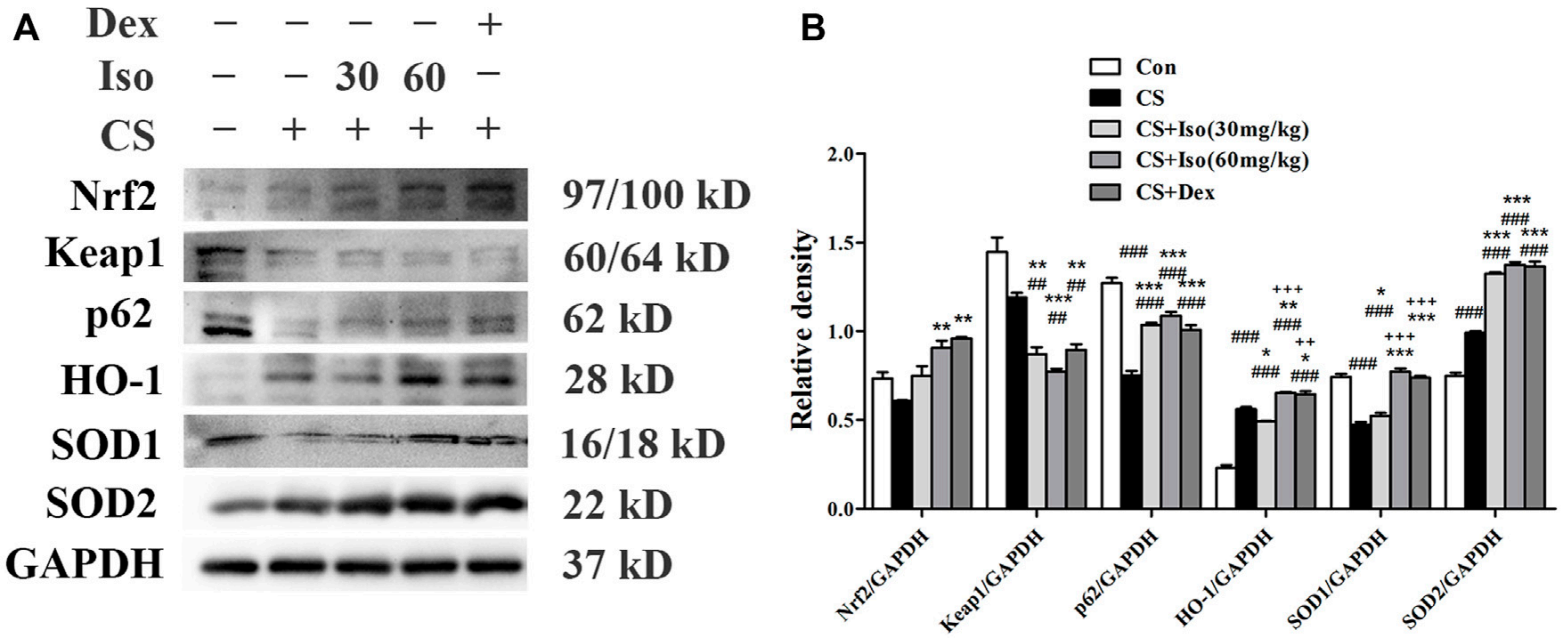
Iso regulates the Nrf2/Keap1 pathway in CS-induced COPD mice. **(A)** The protein expression levels of Nrf2, Keap1, p62, HO-1, SOD1, and SOD2 were measured by western blotting, respectively. GAPDH was used as the internal reference. **(B)** The band intensities of Nrf2, Keap1, p62, HO-1, SOD1, and SOD2 were semiquantified using ImageJ (*n* = 3 mice per group). Data was shown as mean (±SD) **(B)**. To test for group differencses one-way ANOVA **(B)** was applied. ^##^ *p* < 0.01, ^###^ *p* < 0.001 vs. control group, *p < 0.05, **p < 0.01, ***p < 0.001 vs. CS group and ^++^ *p* < 0.01, ^+++^ *p* < 0.001 vs. CS + Iso 30 mg/kg group.

Considering the role of Iso in inhibiting inflammation of COPD, we next explored the underling mechanism. It has been widely reported that the Nrf2/Keap1 pathway plays a major role in the inflammation of COPD (Cho and Kleeberger, 2010; Kubo et al., 2019). As shown in Figure 9, we measured the expression levels of Nrf2, HO-1, superoxide dismutase (SOD)1 and SOD2, and found that Iso treatment obviously enhanced their expression levels in a dose-dependent manner. However, Iso at a dose of 30 mg/kg seems to have little effect on increasing expression of HO-1. Keap1, the negative feedback regulator of Nrf2, was significantly downregulated after Iso treatment. Moreover, it has been reported that p62 regulates the Nrf2/Keap1 pathway by directly interacting with Keap1 to promote degradation of Keap1 by ubiquitination (Lee et al., 2017; Sanchez-Martin et al., 2019). Hence, we further examined the regulatory effect of Iso on p62. As expected, Iso significantly induced p62 accumulation (Figure 9B). These data indicated that Iso activated the Nrf2/Keap1 signaling pathway by promoting p62-associated Keap1 degradation. Moreover, we found that Dex treatment modulated the Nrf2/Keap1 pathway, which exerted a regulatory effect that was similar to that of Iso (60 mg/kg).

## Discussion

**FIGURE 10:**
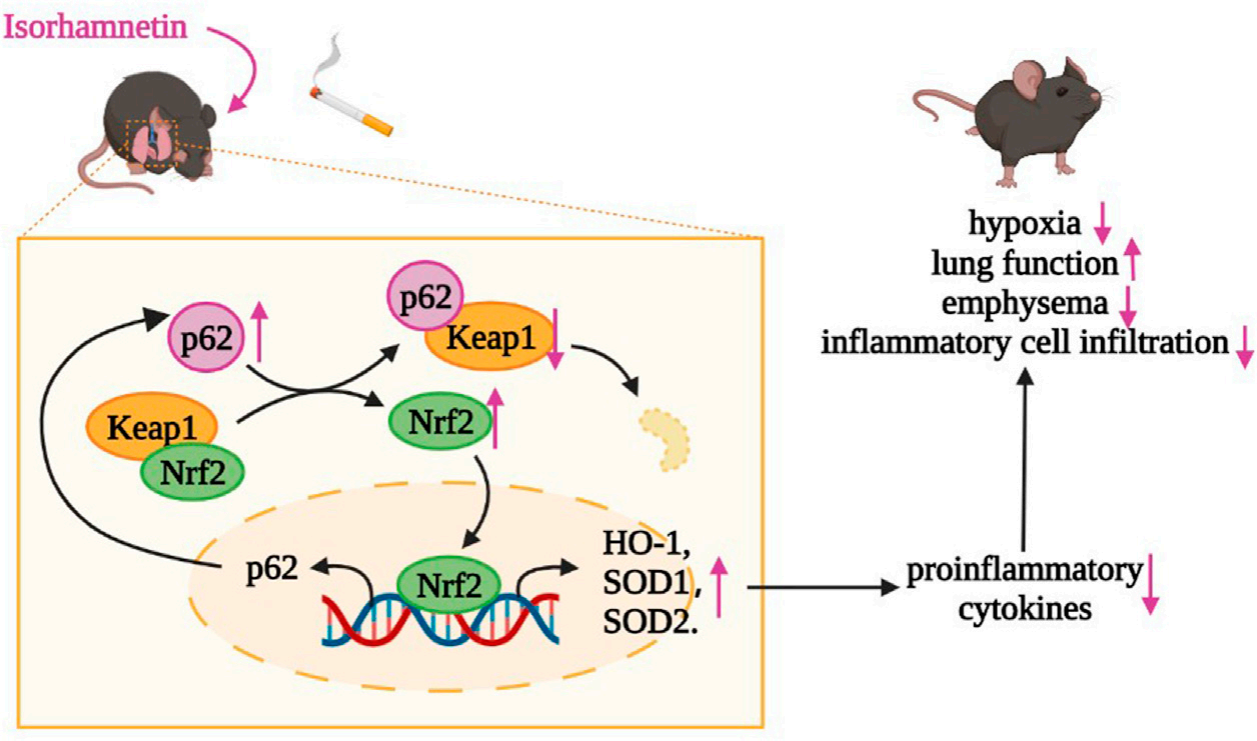
Iso may degrade Keap1 through ubiquitination of p62, thereby activating the Nrf2 pathway to increase the expression of HO-1, SOD1, and SOD2 in lungs of CS-exposed mice, which plays an anti-inflammatory role in COPD.

To our knowledge, this is the first study to investigate the therapeutic effect of Iso on COPD. Our results showed that Iso treatment ameliorated systemic symptoms and improved lung functions by significantly reducing inflammatory cell infiltration, oversecretion of proinflammatory cytokines, and the extent of emphysema and collagen deposition in the lungs of CS-induced COPD mice in a dose-dependent manner. Moreover, Iso reduces the increase of T lymphocytes in peripheral blood of COPD mice. The underlying mechanism of Iso treatment for COPD might be modulation of the Nrf2/Keap1 pathway (Figure 10).

Treatment with Iso significantly ameliorated lung function decline as effectively as Dex treatment, whereas Iso showed no observable side effects compared with the weight loss caused by Dex. Lung function tests are considered to be the most important auxiliary examination in the diagnosis of COPD because they reflect lesions in small airways and are more sensitive than morphological changes of lungs (Rinaldi et al., 2012). Our data suggested that CS-exposed mice exhibited significant airflow restriction and pulmonary hyperinflation as seen by the decreased FEV0.1/FVC value and increased FVC, RI, and Cchord. These data further confirmed the reliability of our model. As a result of impaired lung functions, the mice showed significant symptoms of hypoxia with compensatory increases in the RBC count and hematocrit (Martinez-Bello et al., 2012). Furthermore, systemic symptoms such as weight loss were present in the CS group. Impressively, we observed an apparent improvement of impaired lung functions, thereby reducing hypoxia and weight loss in CS-induced COPD mice following Iso treatment in a dose-dependent manner. However, our data appeared to be contradictory. Dex treatment significantly improved lung functions in CS-induced COPD mice, but did not decrease the compensatory elevation of the RBC count and hematocrit. This phenomenon does not indicate that Dex exerted no effect to improve hypoxia, but may be related to the unique effect of Dex on RBCs (Giglio et al., 1980; Malgor et al., 1987; Tonolo et al., 1988). Moreover, mice weight changes were extremely slow and even showed a downward trend following Dex treatment. These results may be associated with decreased protein synthesis, increased decomposition, and slow growth of muscle and other tissues with long-term glucocorticoid application (Liu et al., 2016; Delanogare et al., 2020).

Iso treatment (especially at 60 mg/kg) markedly attenuated the infiltration of inflammatory cells, secretion of proinflammatory cytokines, and lung pathological injury compared with the control group, which was as effective as dexamethasone treatment. Abnormally activated epithelial cells, monocytes/macrophages, and neutrophils are regarded as the core of the inflammatory response to COPD. They produce proinflammatory mediators such as IL-6, MCP-1 and RANTES. In turn, induced inflammatory cells accumulate in the lungs, which leads to a sustained inflammatory state and thus causes destruction of the alveolar structure, resulting in the decline of lung functions (Bradford et al., 2017; Guan et al., 2020). IL-6, which is primarily secreted by macrophages, has neutrophil chemotactic effects and is associated with COPD severity, the rate of decline in lung functions, and emphysema progression as assessed by CT scans (Bradford et al., 2017). Additionally, IL-6 as a marker of systemic inflammation is associated with some of the systemic manifestations of COPD (Barnes, 2008; Larsson, 2008). MCP-1, which is mainly produced by endothelial cells and alveolar macrophages, is closely associated with induction of chemotaxis in monocytes and a subset of T lymphocytes (Henrot et al., 2019). RANTES is primarily produced by T lymphocytes. The expression levels of RANTES and MCP-1 were increased in BALF of COPD patients, which are associated with an increased influx of CD8^+^ lymphocytes into the airway (Brozyna et al., 2009). Interestingly, MCP-1 and RANTES are also critically involved in enhancing collagen fiber deposition (Russo et al., 2011; Han et al., 2021). Additionally, COX-2 as a main mediator of inflammation has been shown to correlate with inflammatory pathologies of COPD (Ferrer et al., 2019). Therefore, these specific cytokines and chemokines were selected to elucidate the effect of Iso on inflammation in COPD. Our results showed that the total number of leukocytes was significantly increased in BALF of mice in the CS group, the primarily components of which were neutrophils and macrophages. The expression of IL-6, MCP-1, and RANTES in BALF and COX-2 in lung tissue was also significantly increased compared with the control group. However, administration of Iso reduced the increases in the total number of leukocytes, which included neutrophils and macrophages, proinflammatory cytokine secretion, and COX-2 expression, thereby alleviating the damage to the lung structure caused by emphysema and collagen deposition. Additionally, we considered that Iso significantly inhibited CS-induced inflammatory cell accumulation might result from restriction of the chemotaxic effect mediated by MCP-1 and RANTES. Therefore, the mechanism by which Iso regulated MCP-1 and RANTES production warrants further study. More importantly, our results showed that the improvement of all indicators by Iso was not less than that of Dex treatment, which indicates its great potential as a candidate drug for COPD.

Both Iso and Dex treatments significantly attenuated the over-recruitment of CS-induced peripheral blood lymphocytes in mice, while Dex has little effect on T lymphocyte. Many studies have shown that CS exposure elicits adaptive immunity in addition to activating the host innate immune system (Zhu et al., 2009). The imbalance of circulating T lymphocytes in COPD patients in promoting the pathogenesis of COPD airway inflammation has been demonstrated in various studies (Barcelo et al., 2008; Chen et al., 2012). Abnormally activated T lymphocytes secrete cytokines that induce the production of various proinflammatory mediators. In turn, these mediators recruit other immune cells and thus amplify inflammation. However, T lymphocytes also mediate cell death directly *via* the production of cytotoxic mediators and secretion of Fas (Grumelli et al., 2004; Gadgil and Duncan, 2008). Therefore, regulation of T lymphocytes in COPD patients appears to be an effective method to inhibit inflammation. Our results showed increased ratios of lymphocytes and CD3^+^ lymphocytes (mature T lymphocytes) in peripheral blood of COPD mice, which is consistent with previous studies. Impressively, Iso treatment significantly downregulated elevated lymphocytes and T lymphocytes in COPD mice. RANTES, which is secreted mainly by T lymphocytes, is a potent activator and chemokine of T lymphocytes (Turner et al., 1995; Mikolajczyk et al., 2016). However, the level of RANTES induced by CS was decreased by Iso, which suggested that the regulatory effect of Iso on T lymphocytes might be related to RANTES inhibition. Interestingly, Dex treatment had almost no effect on circulating T lymphocytes in COPD mice. This phenomenon may be due to the hormone desensitization of lymphocytes induced by CS exposure (Kaur et al., 2012).

Some studies have shown that Iso exerts anti-inflammatory effects by inhibition of NF-κB, MAPK, PI3K/AKT signaling pathway and reduction of inflammatory factors release (Li et al., 2016; Ren et al., 2021). In particular, a recent study reported that total flavonoids from sea buckthorn (quercetin: 92.423%, isorhamnetin: 3.559%) reduce the expression of proinflammatory factors by blocking the activation of ERK, Akt, and PKCα in mouse models of chronic bronchitis (Ren et al., 2019). Moreover, Iso has been reported to protect cells from oxidative stress through modulating the Nrf2/HO-1 pathway (Choi, 2016). These phenomena led us to investigate the effect of Iso on Nrf2 activating in COPD mice. The role of the Nrf2/Keap1 signaling pathway in COPD airway inflammation has been extensively demonstrated. An increasing number of studies have reported that activation of Nrf2 has a positive effect on the treatment of airway inflammation in COPD (Park et al., 2017; Sun et al., 2019). Nrf2 activation not only alleviates the inflammatory response of COPD by inducing HO-1 and SOD (Park et al., 2017; Sun et al., 2019), but also prevents transcriptional upregulation of proinflammatory mediators that include IL-6 induced by LPS (Kobayashi et al., 2016). Additionally, Nrf2-Keap1 is a primary intracellular antioxidant defense system. Numerous studies have demonstrated that upregulation of Nrf2 inhibits CS-induced ROS and thus inhibits airway inflammation in COPD (Dang et al., 2020). There is ample evidence that targeting the Nrf2/Keap1 signaling pathway may be effective to alleviate COPD airway inflammation (Abed et al., 2015). Iso treatment increased the level of Nrf2 by protecting Nrf2 from Keap1 degradation. More importantly, Iso treatment also upregulated Nrf2 downstream protective factors, such as HO-1, SOD1, and SOD2, which may explain the anti-inflammatory effect of Iso on CS-induced COPD. p62, which was initially identified as 62-kDa protein 9, is a multifunctional signaling pivot, because it has numerous functional domains and participates in activation of the Nrf2/Keap1 pathway (Sanchez-Martin et al., 2019). An increasing number of studies suggest that p62 links the ubiquitin–proteasome system and Nrf2 signaling as described by binding to ubiquitinated Keap1, which inhibits the degradation of Nrf2 and further activates the Nrf2 pathway (Liu et al., 2016; Pan et al., 2016; Tan et al., 2021). Additionally, Nrf2 positively regulates the expression level of p62, which suggests a positive feedback loop (Ichimura et al., 2013). To further explore the mechanism of Iso in activation of Nrf2, we measured the protein expression level of p62. Our results illustrated that Iso treatment upregulated p62 expression, which indicated that the mechanism of Iso in activation of Nrf2 might be connected to the degradation of Keap1 through ubiquitination of p62. However, further studies are needed to confirm the relationship between p62 and Nrf2 in the anti-inflammatory activity of Iso.

There were some limitations in our research, such as not further studying whether the regulation of Iso in Nrf2 signals plays an anti-inflammatory role by improving oxidative stress. However, our study did demonstrate that Iso exhibited anti-inflammatory effects comparable with Dex in COPD and we did not observe discernible side effects of Iso. The high safety profile of Iso may make it a potential drug candidate for COPD.

## Conclusion

Our study indicates that Iso significantly inhibits the inflammatory response in CS-induced COPD mice, mainly by affecting the Nrf2/Keap1 pathway.

## Data Availability

The original contributions presented in the study are included in the article/Supplementary Material, further inquiries can be directed to the corresponding author.
