# Supplementary material for: Isorhamnetin Alleviates Airway Inflammation by Regulating the Nrf2/Keap1 Pathway in a Mouse Model of COPD
Source: Front Pharmacol. 2022 Mar 24;13:860362. doi: 10.3389/fphar.2022.860362 (PMC8988040; doi:10.3389/fphar.2022.860362)
Supplement: Supplementary file 1 [file DataSheet2.PDF]

Supplementary Material

Supplementary Figures

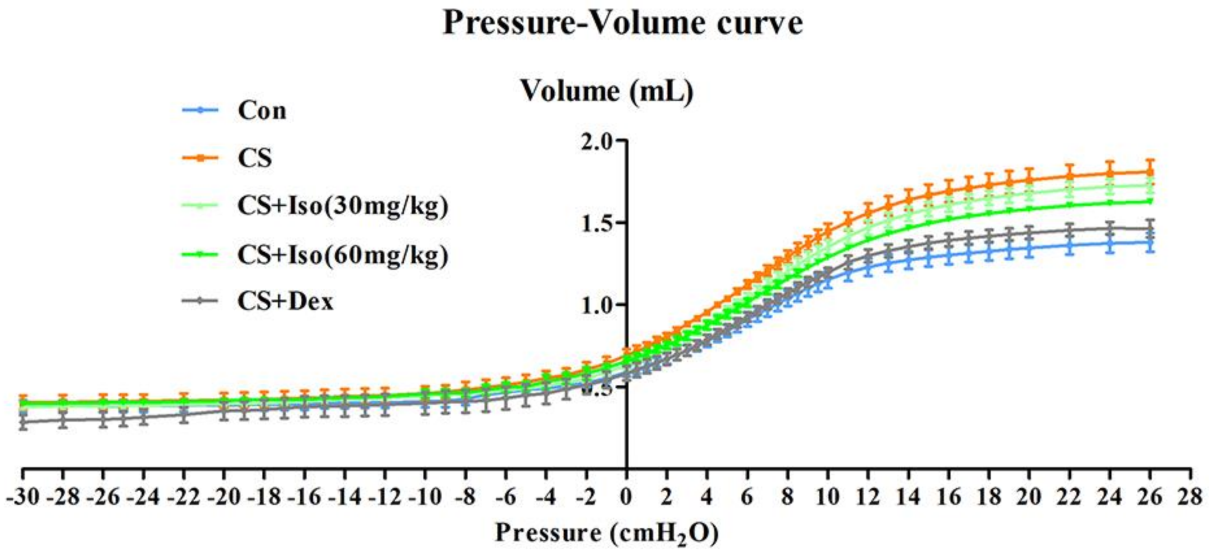

Supplementary Figure 1. CS-induced COPD mice showed a pressure-volume curve that shifting upward and leftwards, which indicated an increase in Cchord (the slope of the P-Vcurve between 0 and +10 cm H_2O pressure).

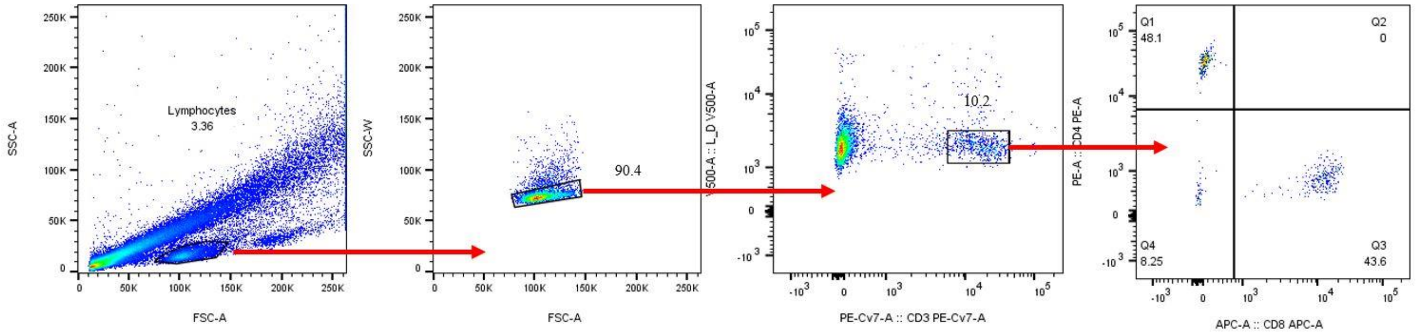

Supplementary Figure 2. The gating strategy for identifying $CD3^+$, $CD3^+CD4^+$ and $CD3^+CD8^+$ lymphocytes.
