# Supplementary figures and images for: Isorhamnetin Alleviates Airway Inflammation by Regulating the Nrf2/Keap1 Pathway in a Mouse Model of COPD
Source: Front Pharmacol. 2022 Mar 24;13:860362. doi: 10.3389/fphar.2022.860362 (PMC8988040; doi:10.3389/fphar.2022.860362)

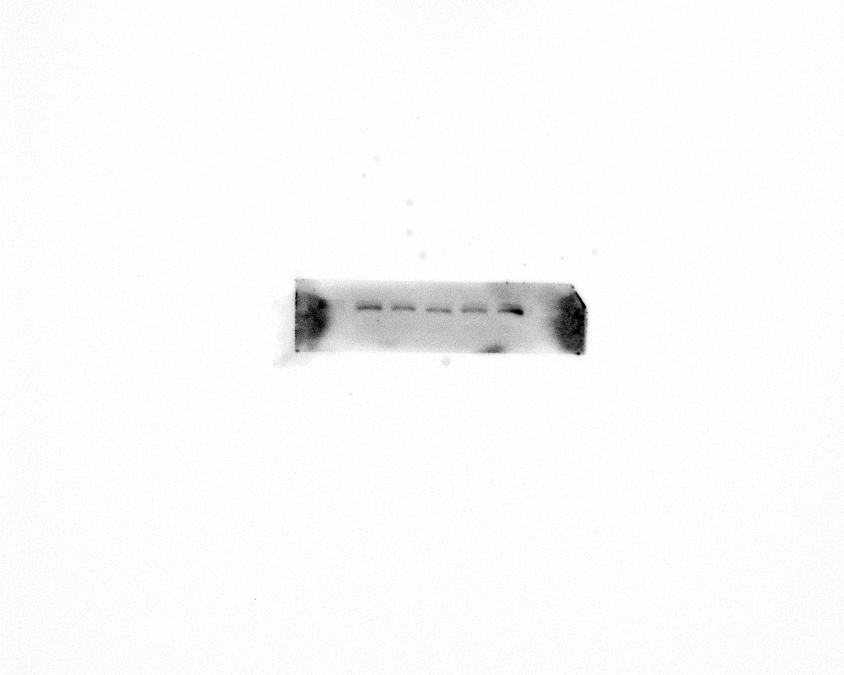

Supplement: Supplementary file 2 [file DataSheet1.ZIP › WB/COX-2/COX-2 (1).tif]

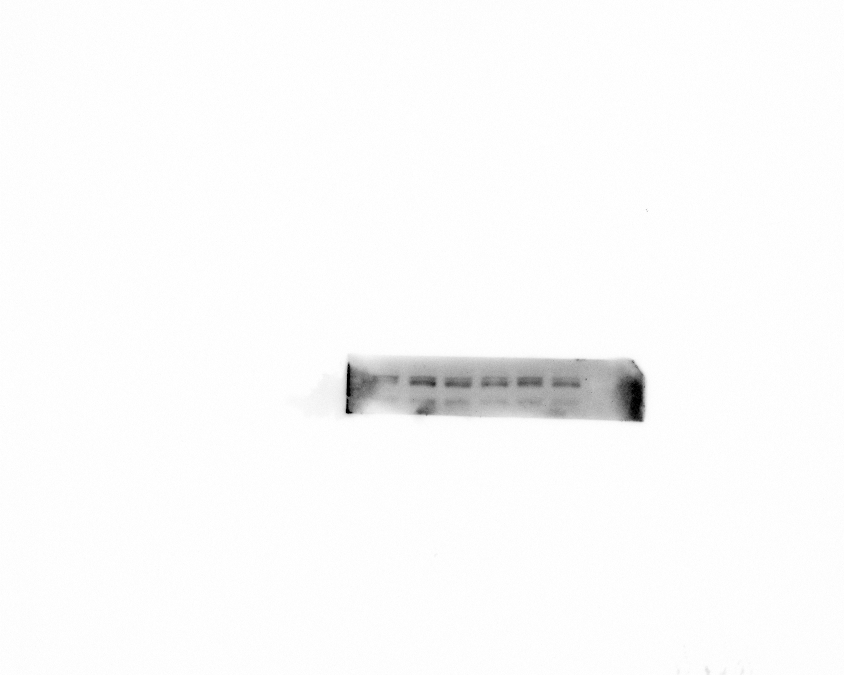

Supplement: Supplementary file 2 [file DataSheet1.ZIP › WB/COX-2/COX-2 (2).tif]

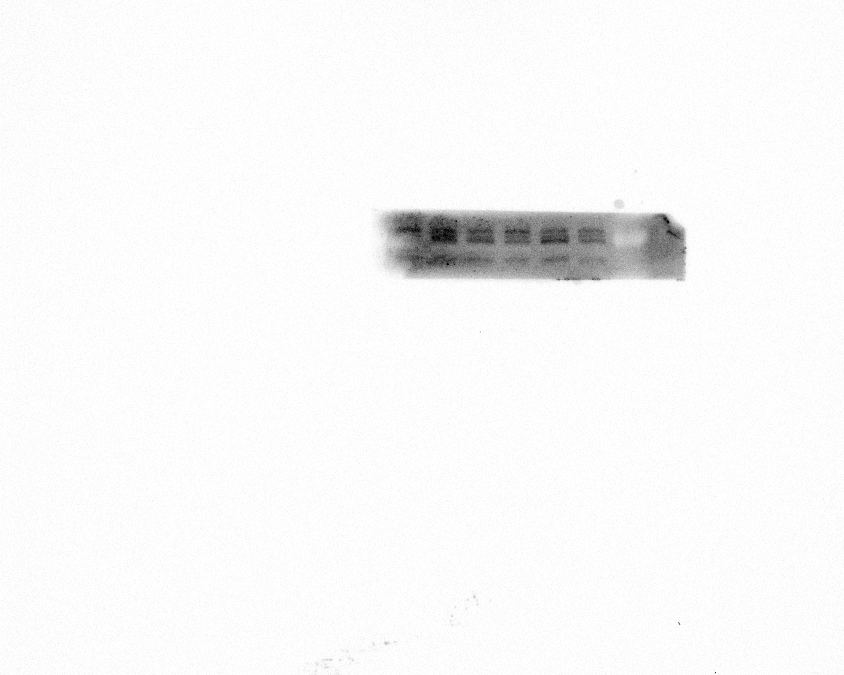

Supplement: Supplementary file 2 [file DataSheet1.ZIP › WB/COX-2/COX-2 (3).tif]

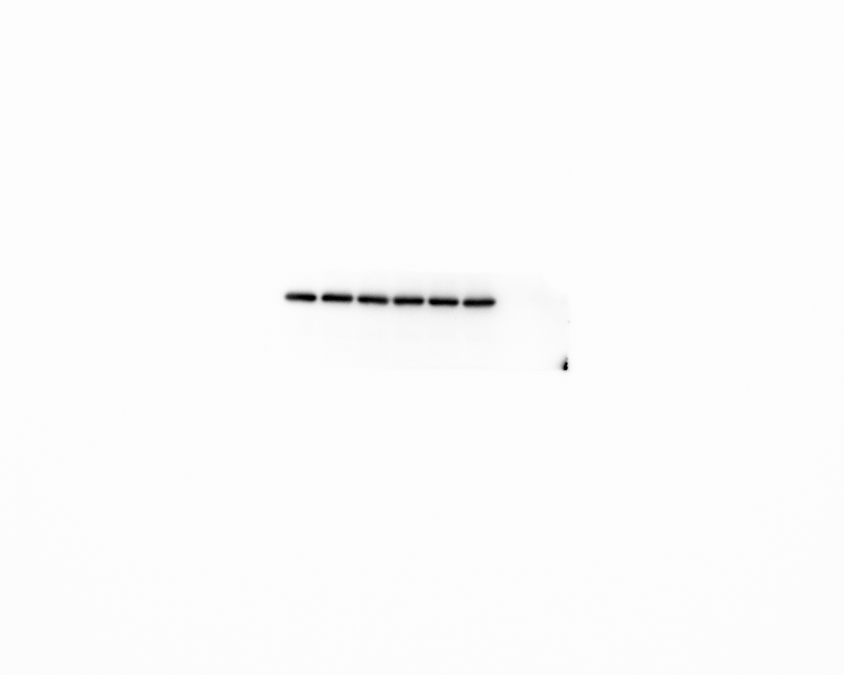

Supplement: Supplementary file 2 [file DataSheet1.ZIP › WB/GAPDH/GAPDH (1).tif]

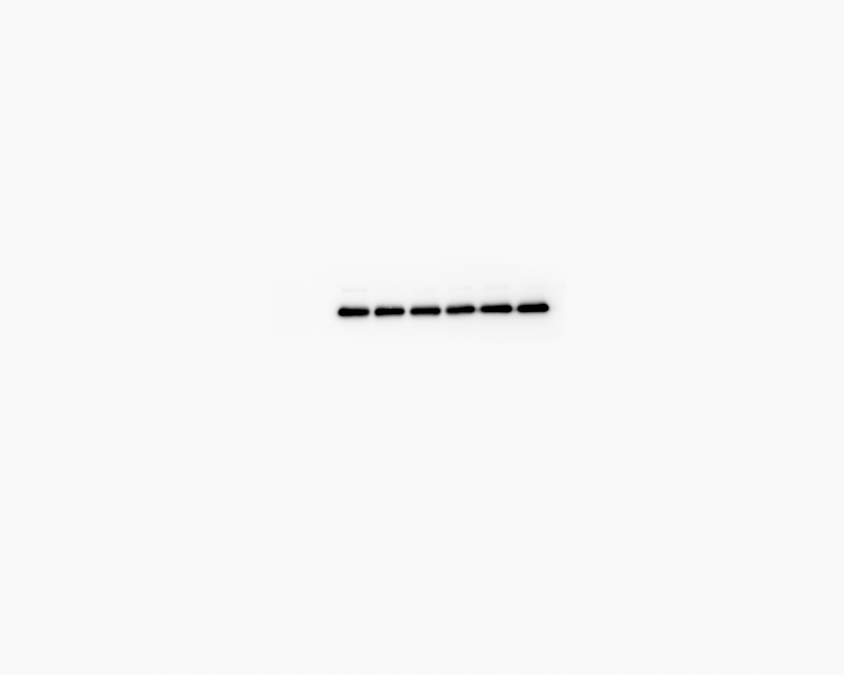

Supplement: Supplementary file 2 [file DataSheet1.ZIP › WB/GAPDH/GAPDH (2).tif]

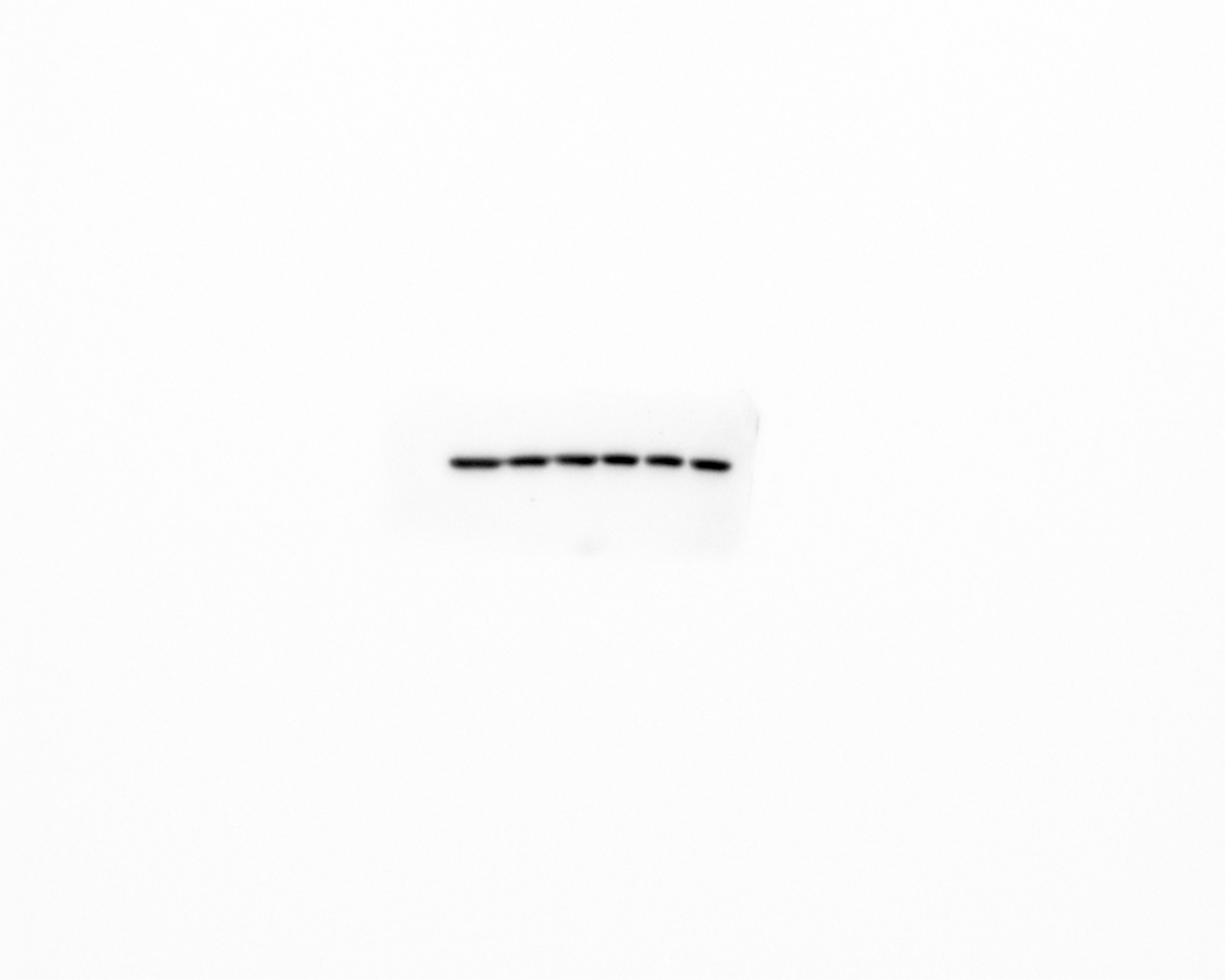

Supplement: Supplementary file 2 [file DataSheet1.ZIP › WB/GAPDH/GAPDH (3).tif]

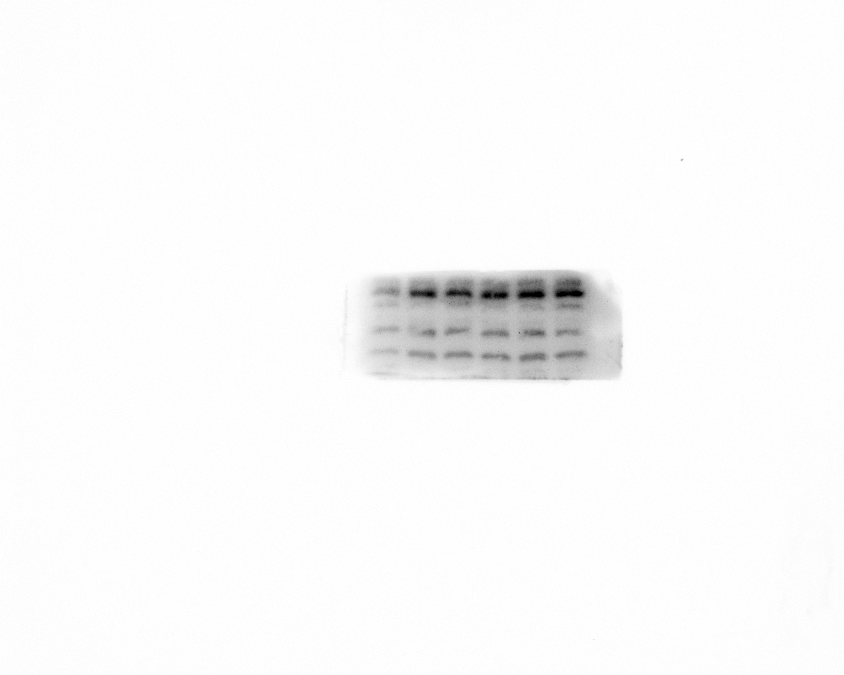

Supplement: Supplementary file 2 [file DataSheet1.ZIP › WB/HO-1/HO-1 (3).tif]

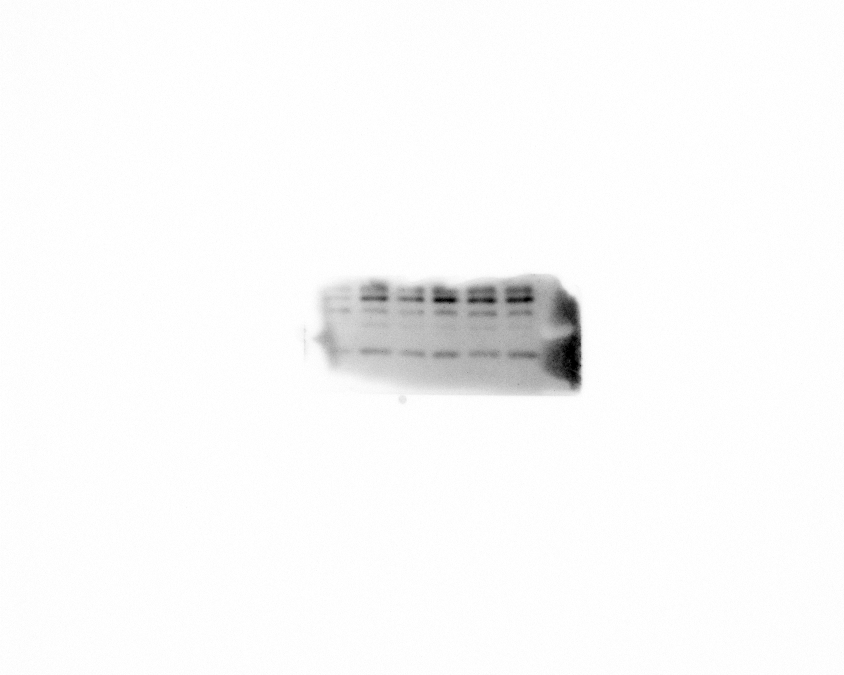

Supplement: Supplementary file 2 [file DataSheet1.ZIP › WB/HO-1/HO-1(1).tif]

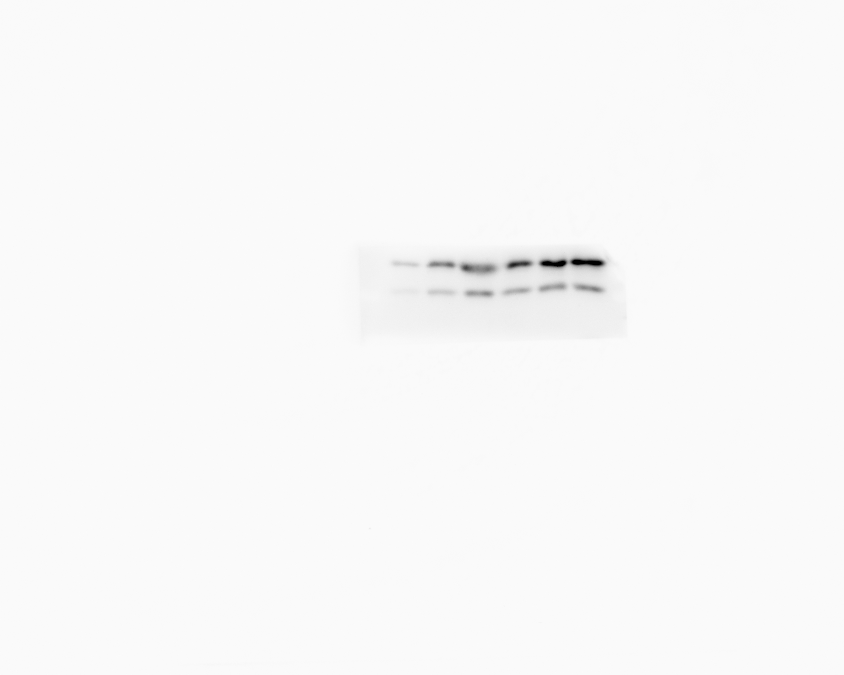

Supplement: Supplementary file 2 [file DataSheet1.ZIP › WB/HO-1/HO-1(2).tif]

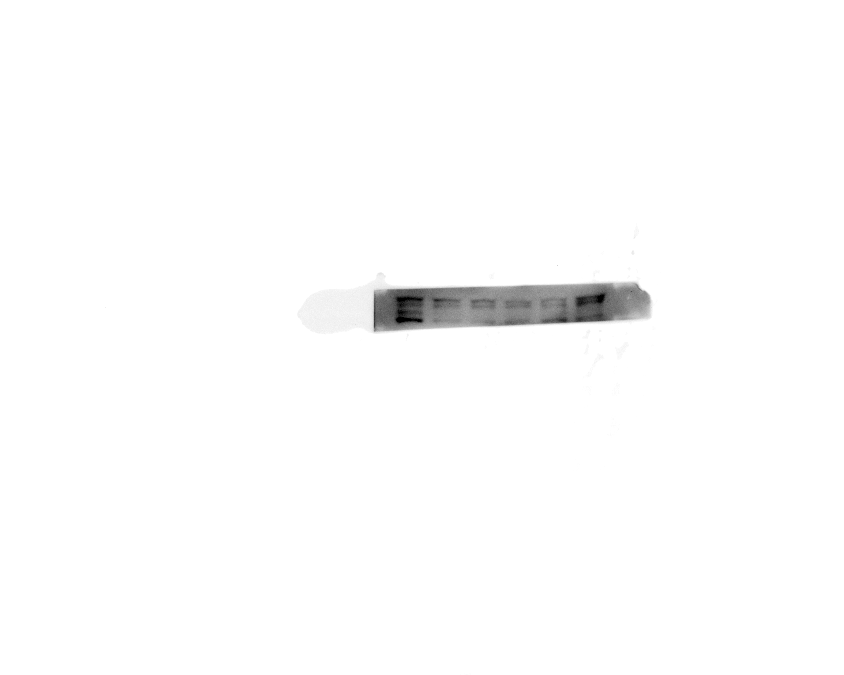

Supplement: Supplementary file 2 [file DataSheet1.ZIP › WB/Keap1/Keap1 (1).tif]

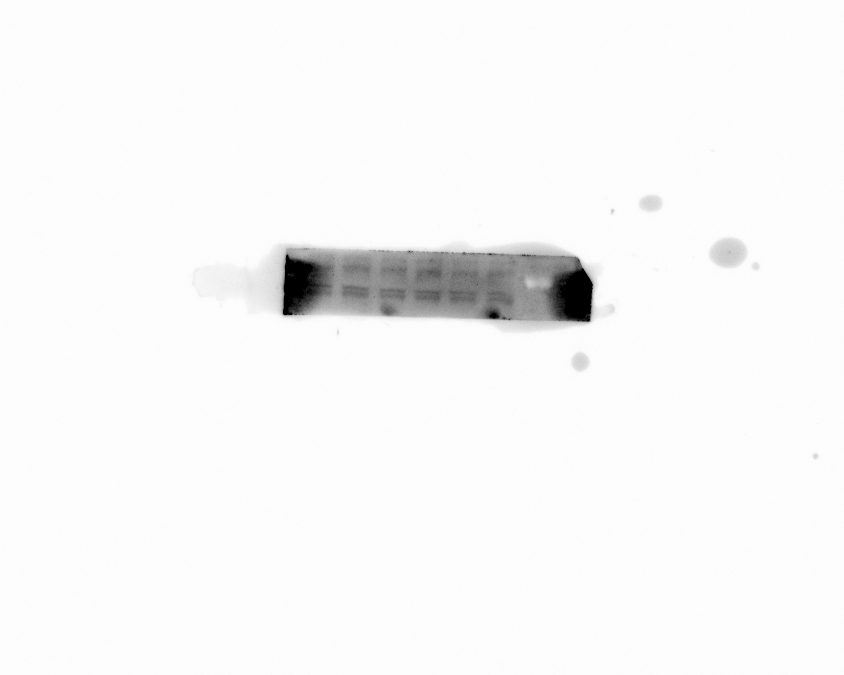

Supplement: Supplementary file 2 [file DataSheet1.ZIP › WB/Keap1/Keap1 (2).tif]

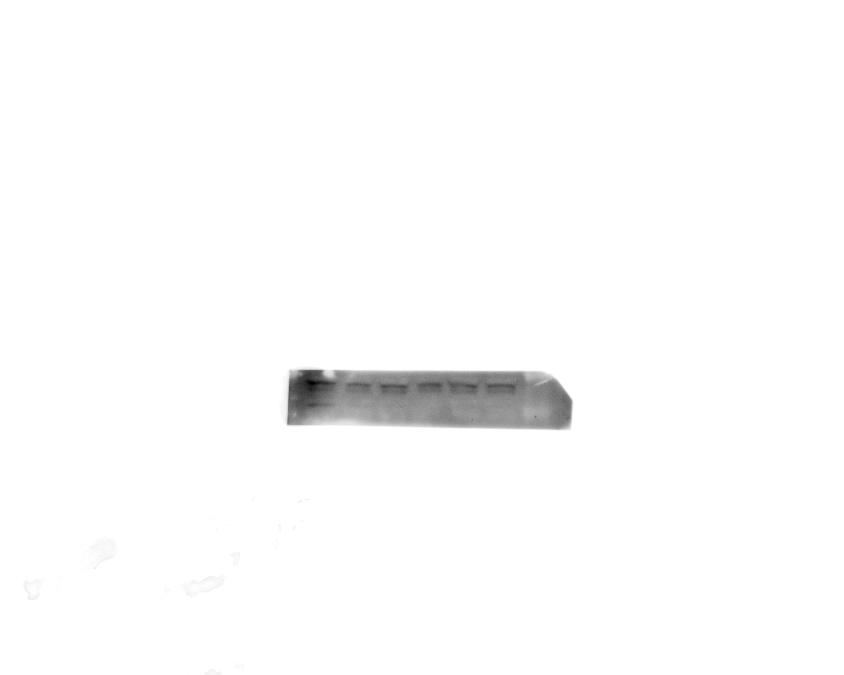

Supplement: Supplementary file 2 [file DataSheet1.ZIP › WB/Keap1/Keap1 (3).tif]

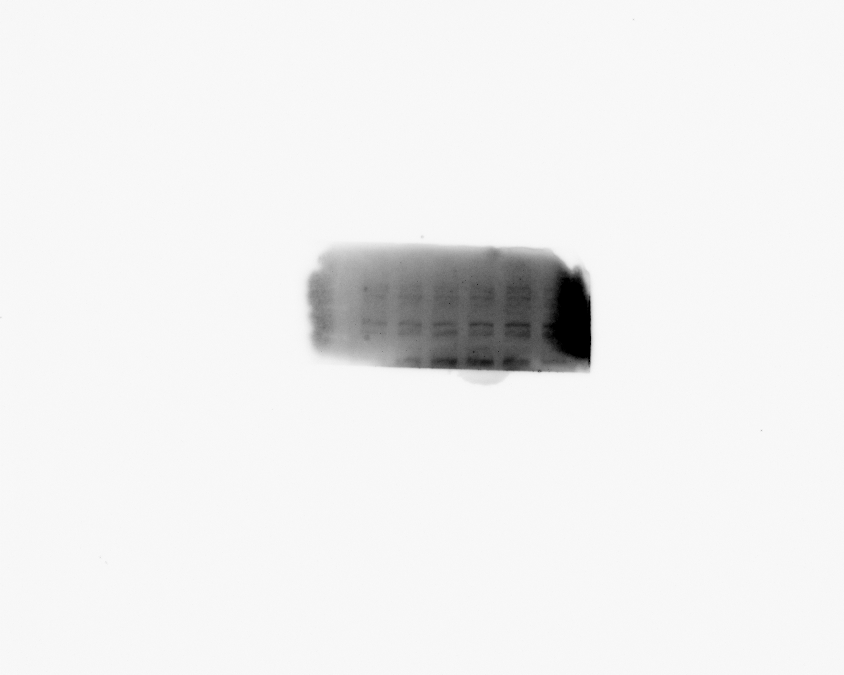

Supplement: Supplementary file 2 [file DataSheet1.ZIP › WB/Nrf2/Nrf2 (1).tif]

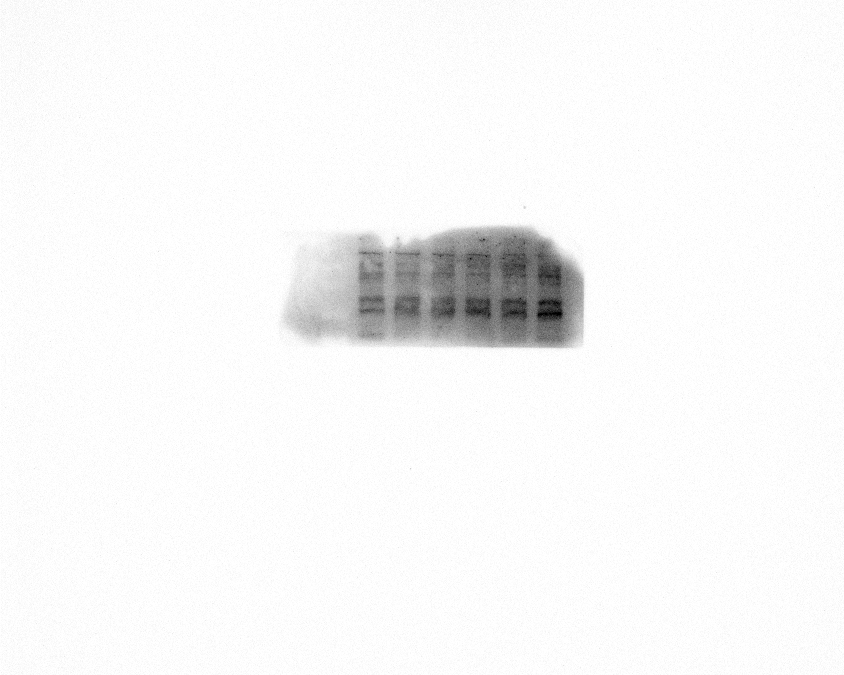

Supplement: Supplementary file 2 [file DataSheet1.ZIP › WB/Nrf2/Nrf2 (2).tif]

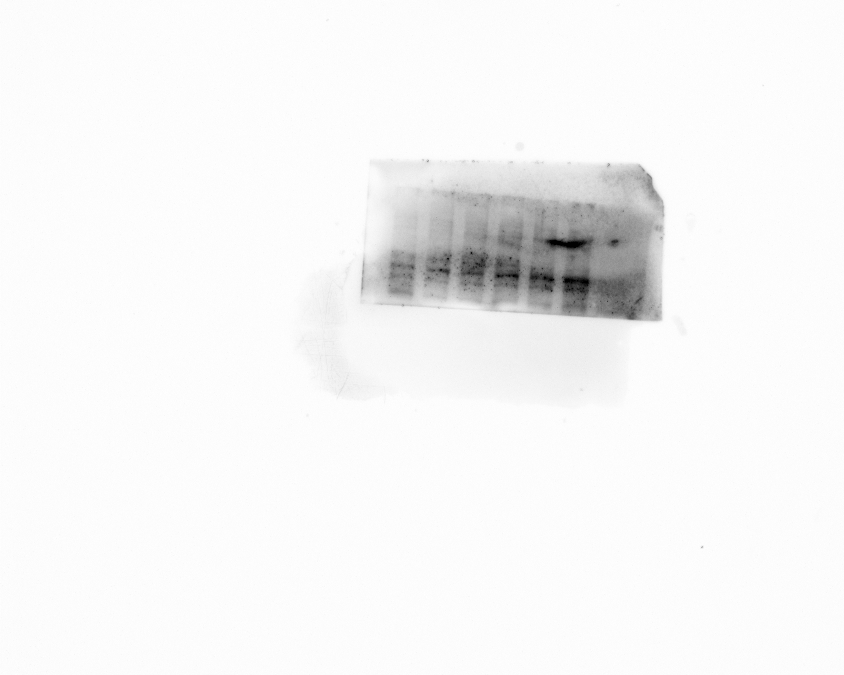

Supplement: Supplementary file 2 [file DataSheet1.ZIP › WB/Nrf2/Nrf2 (3).tif]

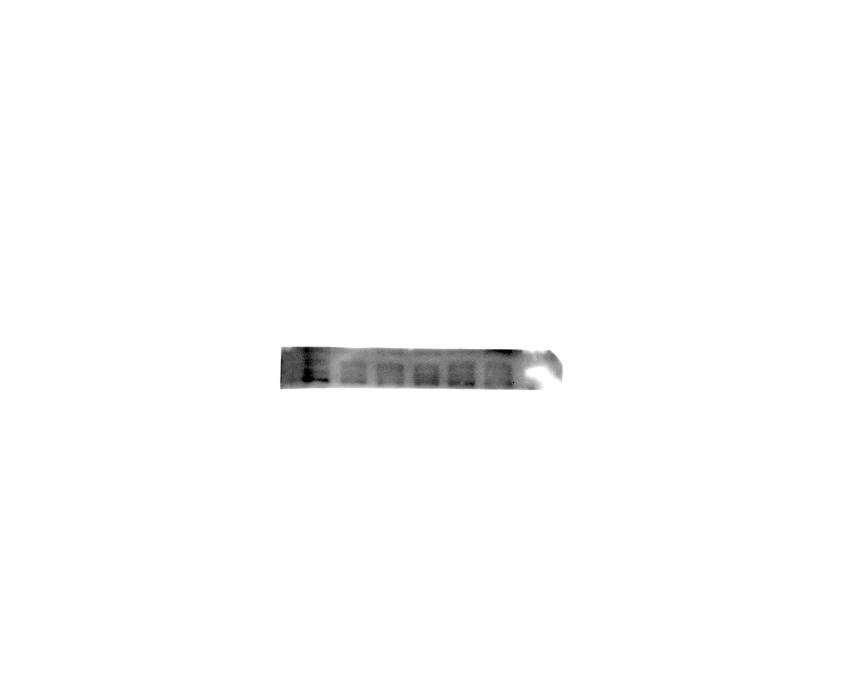

Supplement: Supplementary file 2 [file DataSheet1.ZIP › WB/p62/P62 (2).tif]

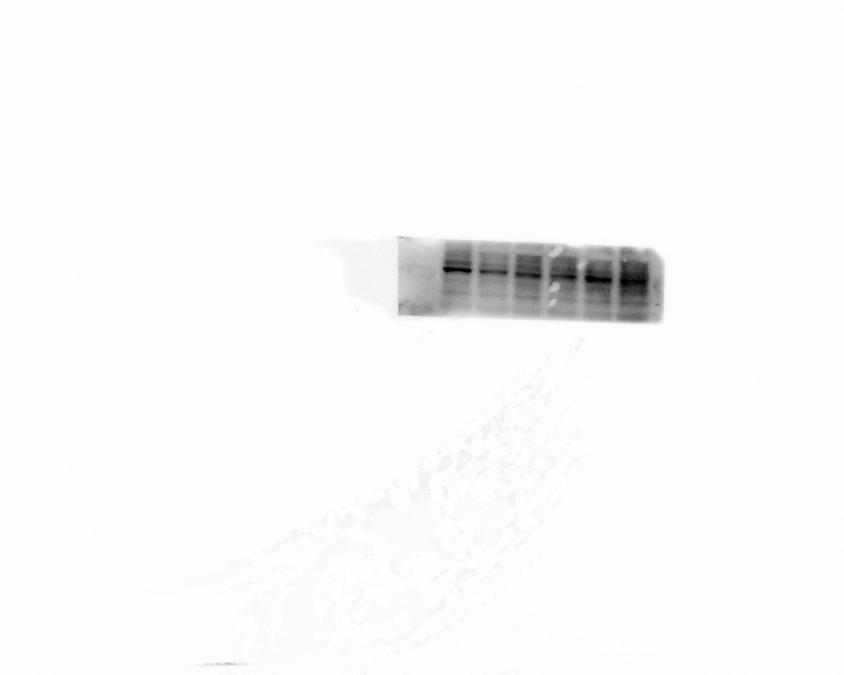

Supplement: Supplementary file 2 [file DataSheet1.ZIP › WB/p62/p62 (3).tif]

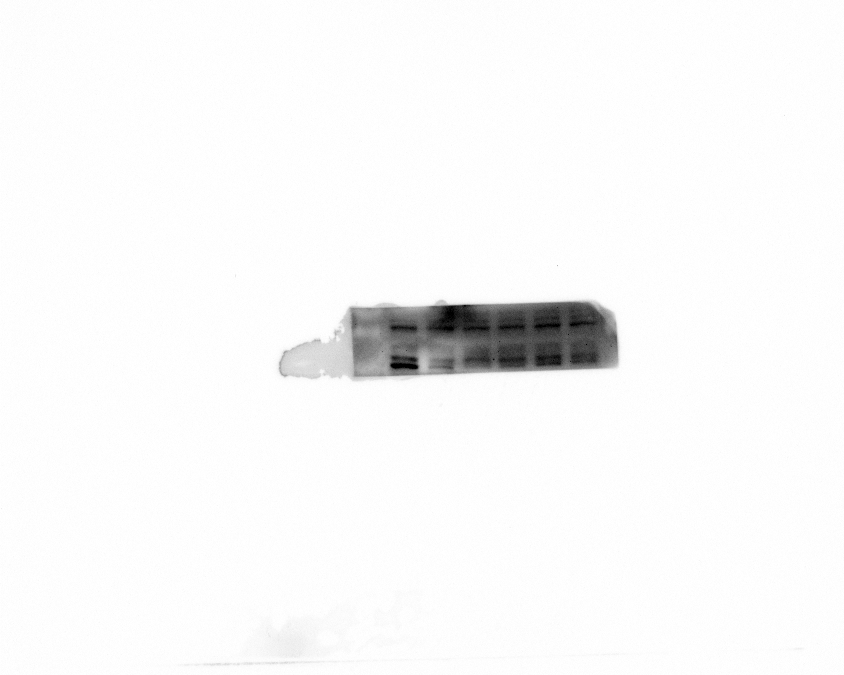

Supplement: Supplementary file 2 [file DataSheet1.ZIP › WB/p62/p62(1).tif]

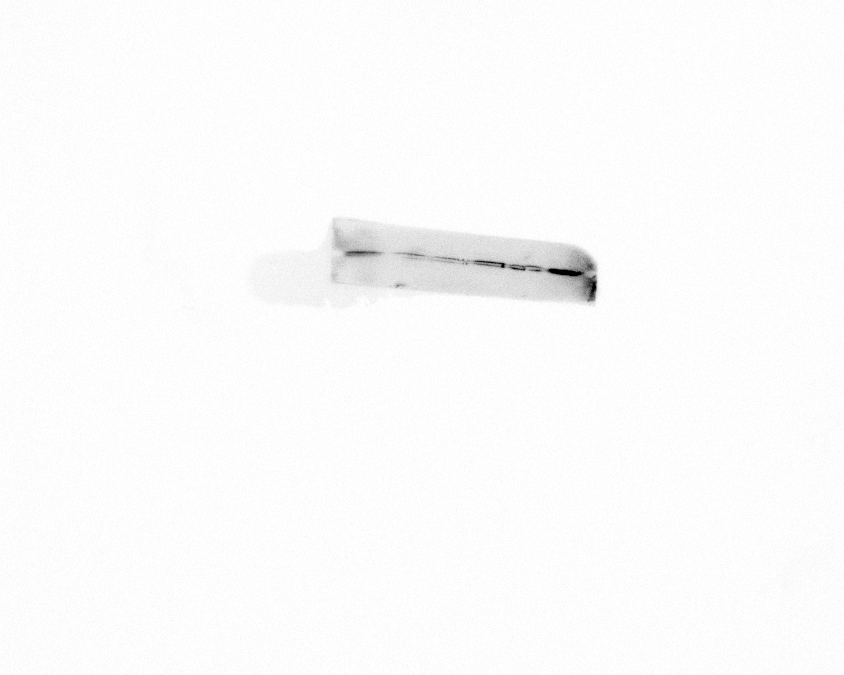

Supplement: Supplementary file 2 [file DataSheet1.ZIP › WB/SOD1/SOD1(1).tif]

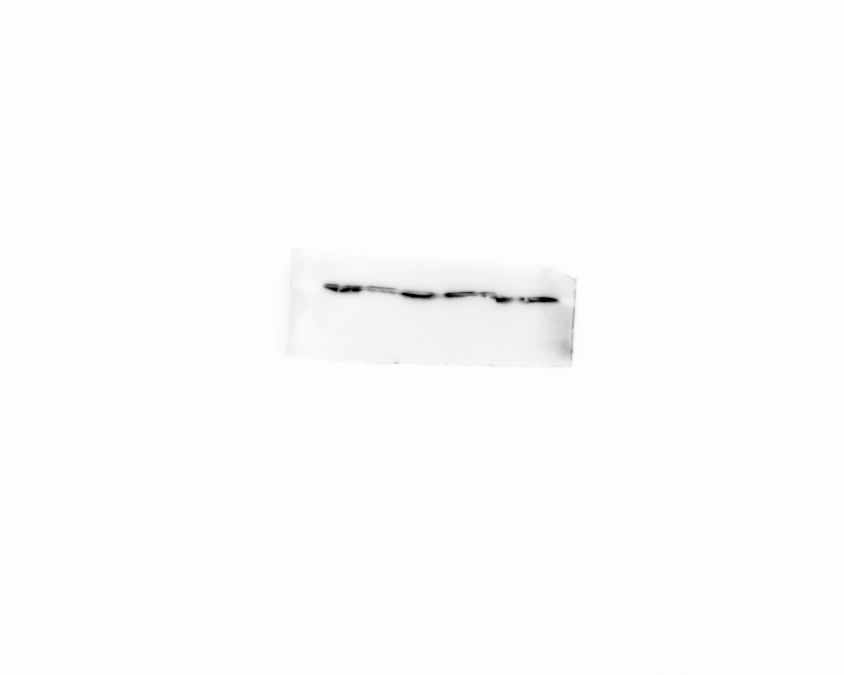

Supplement: Supplementary file 2 [file DataSheet1.ZIP › WB/SOD1/SOD1(2).tif]

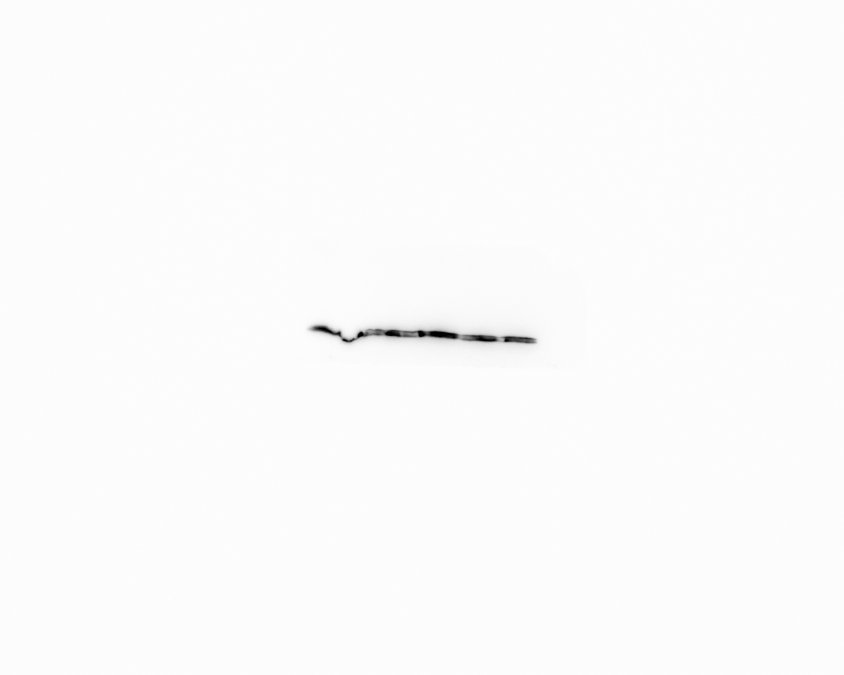

Supplement: Supplementary file 2 [file DataSheet1.ZIP › WB/SOD1/SOD1(3).tif]

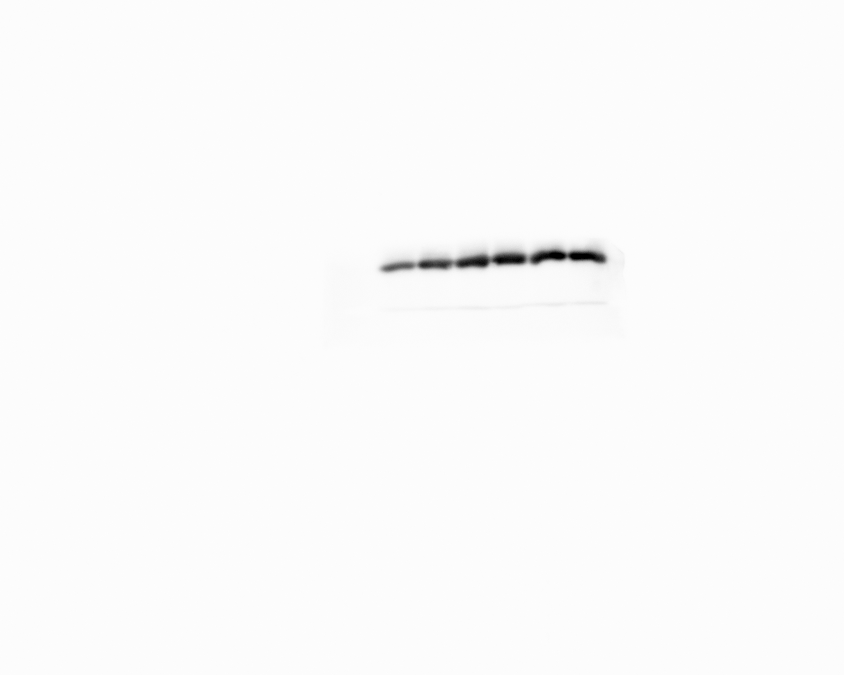

Supplement: Supplementary file 2 [file DataSheet1.ZIP › WB/SOD2/SOD2(1).tif]

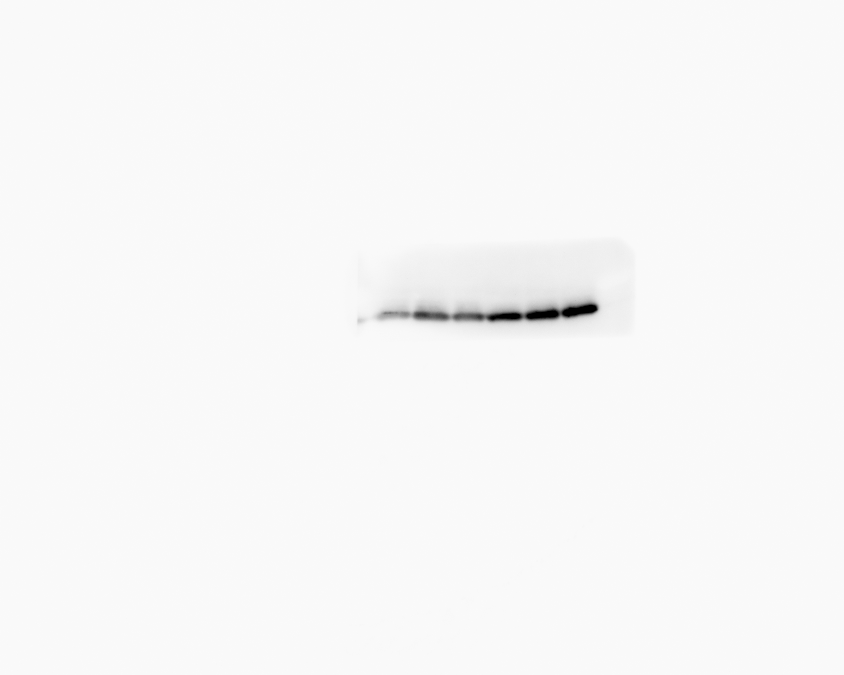

Supplement: Supplementary file 2 [file DataSheet1.ZIP › WB/SOD2/SOD2(2).tif]

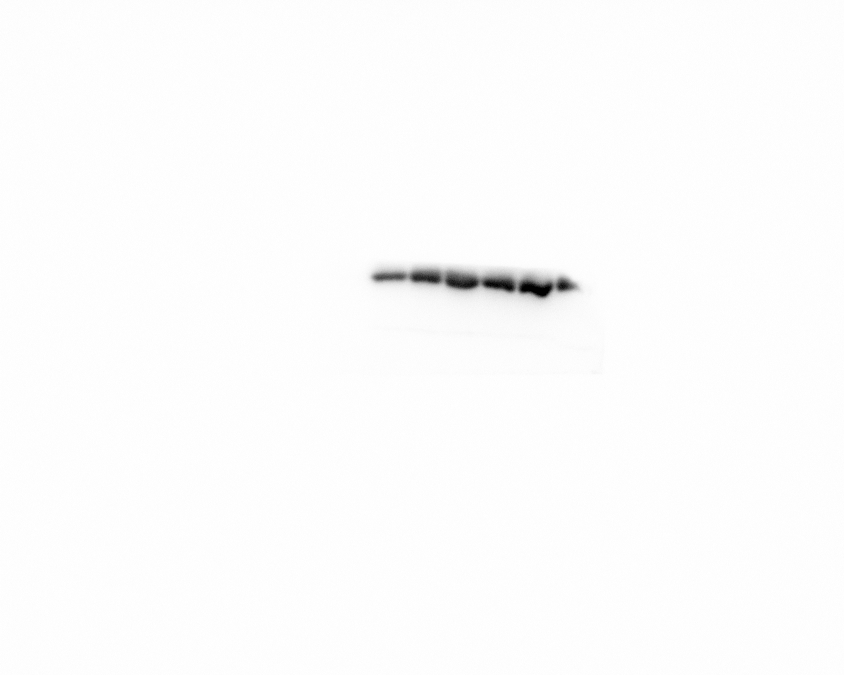

Supplement: Supplementary file 2 [file DataSheet1.ZIP › WB/SOD2/SOD2(3).tif]
